# Ex vivo expansion and hydrogel-mediated in vivo delivery of tissue-resident memory T cells for immunotherapy

**DOI:** 10.1126/sciadv.adm7928

**Published:** 2024-12-13

**Authors:** Shuyi Li, Zhi-Cheng Yao, Hanzhi Wang, Jonathan A. Ecker, Mary O. Omotoso, Jaechan Lee, Jiayuan Kong, Hexiang Feng, Worarat Chaisawangwong, Si-Sim Kang, Sydney R. Shannon, Natalie K. Livingston, Joan G. Bieler, Shweta Singh, Maya L. Zhang, Pilar O’Neal, Emily Ariail, Benjamin Biggs, John W. Hickey, Hai-Quan Mao, Jonathan P. Schneck

**Affiliations:** ^1^ Department of Pathology, Johns Hopkins School of Medicine, Baltimore, MD, USA.; ^2^ Institute for NanoBioTechnology, Johns Hopkins University, Baltimore, MD, USA.; ^3^ Johns Hopkins Translational Immunoengineering Center, Johns Hopkins School of Medicine, Baltimore, MD, USA.; ^4^ Department of Materials Science and Engineering, Whiting School of Engineering, Johns Hopkins University, Baltimore, MD, USA.; ^5^ Translational Tissue Engineering Center, Johns Hopkins School of Medicine, Baltimore, MD, USA.; ^6^ Department of Biomedical Engineering, Whiting School of Engineering, Johns Hopkins University, Baltimore, MD, USA.; ^7^ Department of Biology, Krieger School of Arts and Sciences, Johns Hopkins University, Baltimore, MD, USA.; ^8^ Department of Biomedical Engineering, Johns Hopkins School of Medicine, Baltimore, MD, USA.; ^9^ Department of Chemical and Biomolecular Engineering, Whiting School of Engineering, Johns Hopkins University, Baltimore, MD, USA.; ^10^ Department of Biomedical Engineering, Duke University, Durham, NC, USA.; ^11^ Institute for Cell Engineering, Johns Hopkins School of Medicine, Baltimore, MD, USA.

## Abstract

Tissue-resident memory T (T_RM_) cells preferentially reside in peripheral tissues, serving as key players in tumor immunity and immunotherapy. The lack of effective approaches for expanding T_RM_ cells and delivering these cells in vivo hinders the exploration of T_RM_ cell–mediated cancer immunotherapy. Here, we report a nanoparticle artificial antigen-presenting cell (nano-aAPC) ex vivo expansion approach and an in vivo delivery system for T_RM_ cells. Using the nano-aAPC platform, we expanded functional antigen-specific murine and human T_RM_-like CD8^+^ T cells ex vivo. We also developed an injectable macroporous hyaluronic acid (HA) hydrogel to deliver T_RM_-like cells. T_RM_-like cells delivered in the optimized HA hydrogel trigger robust local and systemic antitumor immunity and show synergistic effects with anti–PD-1 treatment. Our findings suggest that nano-aAPC–induced T_RM_-like cells, coupled with a hydrogel delivery system, offer an efficient way to advance the understanding of T_RM_ cell–mediated cancer therapy.

## Teaser

Tissue-resident memory-like T cells delivered in the hydrogel drive effective local and distal antitumor immunity.

## INTRODUCTION

Tissue-resident memory T (T_RM_) cells preferentially reside in nonlymphoid tissues without recirculation, which differs from recirculating memory T cells (central memory T cells and effector memory T cells) (*1*, *2*). They are frontline, tissue-specific, critical mediators of anti-pathogen immunity and serve as key players in tumor immunity and immunotherapy (*3*–*10*). T_RM_ cells express high levels of adhesion molecule CD103 [αE(CD103)β7], enabling T_RM_ cells to target and accumulate at tumor sites by interacting with E-cadherin. T_RM_ cells can produce high levels of cytotoxic molecules to directly kill target tumor cells and respond more rapidly than circulating memory cells when reexposed to cognate tumor antigens (*11*, *12*).

In addition, T_RM_ cells can trigger innate immune cell and recirculating memory T cell recruitment. They do this by releasing cytokines and chemokines after a local antigen challenge, thus regulating local immunosurveillance (*13*, *14*). Tumor-infiltrating CD8^+^ T cells expressing T_RM_ markers found in biopsies are associated with a favorable prognosis for numerous solid tumors, including melanoma (*15*), breast cancer (*16*), ovarian cancer (*17*), cervical cancer (*18*), and lung cancer (*19*). While many T_RM_ cell characteristics suggest they are highly effective in inhibiting tumor growth, the lack of effective methods to rapidly induce and expand T_RM_ cells is an obstacle to elucidating the role of T_RM_ cells in cancer immunotherapy. Further, the systemic delivery of T_RM_ cells shows limited efficacy, partly due to the poor homing capacities of T_RM_ cells without homing markers sphingosine-1-phosphate receptor 1 (S1PR1), CCR7, and CD62L (*20*–*22*). Thus, an optimal approach to overcoming the limited homing of T_RM_ cells is essential to effective T_RM_ cell–based cancer immunotherapy.

**Fig. 1.**
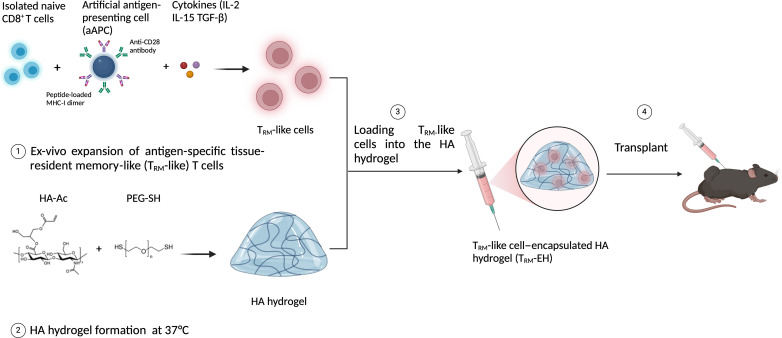
Schematics of T_RM_-like cell generation stimulated and expanded by nano-aAPCs conjugated with pMHC-I and anti-CD28 antibodies in the presence of IL-2, IL-15, and TGF-β, and delivery of T_RM_-like cells by HA hydrogel in vivo. Created with BioRender.com.

Previously, we developed a paramagnetic nanoparticle artificial antigen-presenting cell (nano-aAPC) platform conjugated with signal 1 [peptide-loaded major histocompatibility complex class I (pMHC-I)] and signal 2 (costimulatory molecule, anti-CD28) for antigen-specific T cell activation and expansion (*23*). Nano-aAPC–based stimulation was also supported by using soluble cytokines, signal 3, which direct T cell fate and lineage commitment for both murine and human CD8^+^ T cell expansion (*24*). This approach achieves effective expansion of cognate T cells, comparable to other standard stimulation methods such as peptide-pulsed autologous antigen-presenting cells (APCs) or anti-CD3/anti-CD28 microbeads (*25*, *26*). It also overcomes the challenges associated with the lack of specificity in anti-CD3 stimulation, enabling up to a 1000-fold expansion of antigen-specific CD8^+^ T cells from patients with melanoma in 2 weeks (*27*). The nano-aAPC platform has also been adapted as a high-throughput platform to enrich and expand a range of rare murine or human antigen-specific T cells (*23*). To overcome the induction and expansion challenges associated with generating antigen-specific T_RM_-like cells, we used nano-aAPC conjugated with pMHC-I molecules and anti-CD28 and polarized the antigen-specific T cells into T_RM_ cells by including transforming growth factor–β (TGF-β) and interleukin-15 (IL-15) as signal 3. These are indispensable factors for promoting differentiation and survival of T_RM_ cells (*28*–*30*) as local TGF-β production has been shown to up-regulate CD103 expression (an important T_RM_ cell marker) by activating the Smad2/3 signaling pathway (*31*, *32*). Here, we develop and optimize a T_RM_ cytokine mix consisting of IL-2 [a cytokine signal supporting T cell proliferation; (*33*)], IL-15, and TGF-β to stimulate and rapidly expand antigen-specific murine and human T_RM_-like cells (Fig. 1).

We previously reported an injectable, biodegradable hyaluronic acid (HA) hydrogel–based platform for subcutaneously delivering mesenchymal stem cells with improved cell retention and regenerative outcomes at the tissue repair site (*34*, *35*). HA hydrogel is highly porous, allowing cell infiltration and sustained release. Here, we adopted the HA hydrogel with optimized stiffness and pore size for T_RM_-like cell delivery, termed T_RM_-like cell–encapsulated hydrogel (T_RM_-EH) (Fig. 1), to drive effective local and systemic antitumor immune responses in mouse colon carcinoma and melanoma models and explored the synergistic effect of T_RM_-EH with a programmed cell death protein 1 (PD-1) blocking antibody.

## RESULTS

### Nano-aAPCs stimulate antigen-specific murine CD8^+^ T cells with T_RM_-like phenotype and transcriptional profile

A T cell stimulation system is needed to study the effective cytokine signals for generation of T_RM_-like cells. A variety of standard T cell receptor stimulation approaches can be used for T cell activation, such as nonspecific anti-CD3/anti-CD28 Dynabeads and plate-bound anti-CD3/anti-CD28 proteins, both of which can yield large numbers of activated T cells. We have previously shown that nano-aAPCs can achieve comparable expansion of T cells to these standard approaches (*25*, *26*). To address the lack of specificity in anti-CD3 stimulation (*36*), we opted to use nano-aAPCs, composed of dextran-coated iron oxide nanoparticles, chemically conjugated with a pMHC-I molecule, MHC-I H-2Kb bound to the ovalbumin-derived peptide, SIINFEKL (Kb-OVA), or MHC-I H-2Db bound to the GP100-derived peptide, KVPRNQDWL (Db-GP100), as signal 1 and costimulatory molecule anti-CD28 as signal 2. The synthesized nano-aAPCs had a slightly increased hydrodynamic diameter of ~80 nm after protein conjugation (fig. S1). Each Kb-OVA/aCD28 bead was conjugated with an average of 47 ± 14 MHC-I Kb-OVA molecules, and each Db-GP100/aCD28 bead had an average of 27 ± 9 MHC-I Db-GP100 molecules conjugated to its surface (fig. S2).

**Fig. 2.**
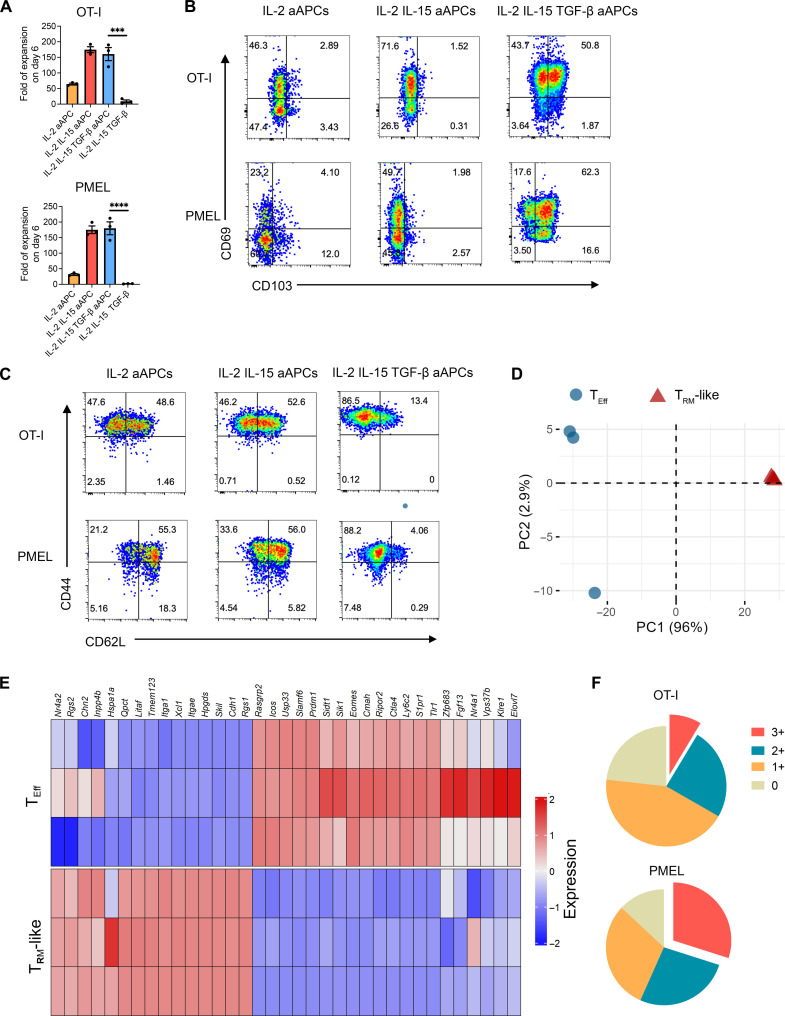
Nano-aAPCs with IL-2, IL-15, and TGF-β induce functional antigen-specific mouse CD8^+^ T cells with T_RM_ phenotype and transcriptional profile. (**A**) Fold expansion of PMEL and OT-I CD8^+^ T cells expanded by nano-aAPCs in the presence of various cytokine mixes over 6 days. Each bar represents means ± SEM. *n* = 3. One-way analysis of variance (ANOVA) with Tukey’s multiple comparisons test. ***P < 0.001. (**B**) Expression of T_RM_ marker (CD69 and CD103) on PMEL and OT-I CD8^+^ T cells on day 6. (**C**) Expression of homing marker (CD62L) on PMEL and OT-I CD8^+^ T cells on day 6. (**D**) Principal components analysis (PCA) of top 1000 differentially expressed gene (DEG) expression data from OT-I CD8^+^ T cells expanded by aAPCs in the presence of IL-2 (T_Eff_) or IL-2, IL-15, and TGF-β (T_RM_-like). (**E**) Heatmap of T_RM_-related signature genes expression in OT-I CD8^+^ T cells expanded by aAPCs in the presence of IL-2 (T_Eff_) or IL-2/IL-15/TGF-β (T_RM_-like) (**F**) Pie charts showing the polyfunctionality profile of PMEL and OT-I CD8^+^ T cells expanded in the medium containing nano-aAPCs, IL-2, IL-15, and TGF-β. Pie charts represent the fractions of expanded T cells secreting none or any (1, 2, or 3) of the three cytokines IFN-γ, TNF-α, and IL-2.

CD8^+^ T cells isolated from OT-I or PMEL mouse spleens or lymph nodes were incubated with nano-aAPCs in the presence of various cytokine mixes. OT-I or PMEL CD8^+^ T cells (T cells specific to ovalbumin (OVA) or GP100 melanoma antigen) robustly expanded up to 200-fold in the nano-aAPC treatment by day 6 (Fig. 2A). Phenotypic analysis demonstrated that CD8 T cells, PMEL or OT-I, expanded in a cytokine mix containing IL-2, IL-15, and TGF-β expressed high levels of the T_RM_ cell markers CD103 and CD69 and reduced levels of homing marker CD62L compared with those expanded with IL-2 or IL-2/IL-15. This finding is consistent with most T_RM_ subsets (Fig. 2, B and C) (*37*).

We profiled the transcriptomes of OT-I CD8^+^ T cells expanded by nano-aAPCs with IL-2 or IL-2/IL-15/TGF-β by RNA sequencing. The principal component 1 (PC1) and PC2 axes of variation in the principal components analysis (PCA) of the two CD8^+^ T cell subsets effectively distinguished CD8^+^ T cells expanded in IL-2, IL-15, and TGF-β from those expanded solely in IL-2. Notably, the PCA demonstrated a transcriptional distinctiveness of the cells with PC1 accounting for 96% of the variation in gene expression between the two groups (Fig. 2D). In addition, CD8^+^ T cells expanded in IL-2/IL-15/TGF-β revealed a high degree of congruence in the transcriptional profiles to T_RM_ core signature (*38*). Specifically, we observed up-regulation of genes associated with tissue residency, such as *Cdh1*, *Itga1*, *Itgae*, *Rgs1*, and *Rgs2*, in CD8^+^ T_RM_-like cells. In contrast, genes linked with circulating memory CD8^+^ T cells, including tissue-egress factors like *S1pr1*, were notably down-regulated (Fig. 2E). Among the 34 genes constituting the T_RM_ core signature, 23 showed significant up- or down-regulation in cells expanded in IL-2/IL-15/TGF-β compared to those expanded in IL-2 alone.

Further, we conducted Gene Ontology (GO) functional enrichment analysis and gene set enrichment analysis (GSEA) on differentially expressed genes (DEGs) to investigate enriched pathways. Among the up-regulated DEGs in cells expanded in IL-2/IL-15/TGF-β, we observed significant enrichment in biological processes associated with the regulation of T cell activation, immune response, positive regulation of cytokine production, positive regulation of adhesion, and regulation of migration (fig. S3, A and C). Conversely, the down-regulated genes were predominantly linked to the negative regulation of the cell cycle process, DNA replication, nuclear division, and leukocyte cell-cell adhesion (fig. S3, B and D).

These findings indicate that T cells expanded in IL-2, IL-15, and TGF-β may be in a more active state. They may also be better able to produce cytokines, promote adhesion, and reduce migration. Collectively, nano-aAPCs expanded CD8^+^ T cells with IL-2, IL-15, and TGF-β induce a distinct population with a T_RM_-like phenotype and transcriptional profile.

To analyze the functionality of the T_RM_-like OT-I and PMEL cells, we activated cells with phorbol 12-myristate 13-acetate (PMA)/ionomycin, which is known to nonspecifically activate T cells and stimulate cytokine production. We used flow cytometry to analyze cell expression of interferon-γ (IFN-γ), tumor necrosis factor–α (TNF-α), and IL-2. As expected, CD8^+^ T cells expanded in IL-2; IL-2 and IL-15; or IL-2, IL-15, and TGF-β produce substantial amounts of cytokines after restimulation (fig. S4). We further analyzed the polyfunctionality of CD8^+^ T cells expanded in IL-2, IL-15, and TGF-β. For OT-I CD8^+^ T cells, 10% of the stimulated cells secreted all three cytokines IFN-γ, TNF-α, and IL-2 (termed 3+ functional cells). For PMEL cells, up to 30% were 3+ functional cells (Fig. 2F and fig. S5). Moreover, we separately examined the polyfunctionality of nano-aAPC–induced T_RM_ subsets with varying expression of CD103 markers. Compared with CD103^–^ T cells, CD103^+^ T cells exhibited a significantly higher percentage of 3+ functional cells. Specifically, CD103^+^ T cells displayed a 10% higher proportion of 3+ functional cells in PMEL cells and 2.5% higher in OT-I cells. Thus, a higher level of polyfunctionality was associated with CD103^+^ T cells (fig. S6). These data suggest that nano-aAPCs, combined with an optimized cytokine milieu, induce highly functional antigen-specific CD8^+^ T cells with a T_RM_-like phenotype.

### Macroporous HA hydrogels directed the release of T_RM_-like cells

**Fig. 3.**
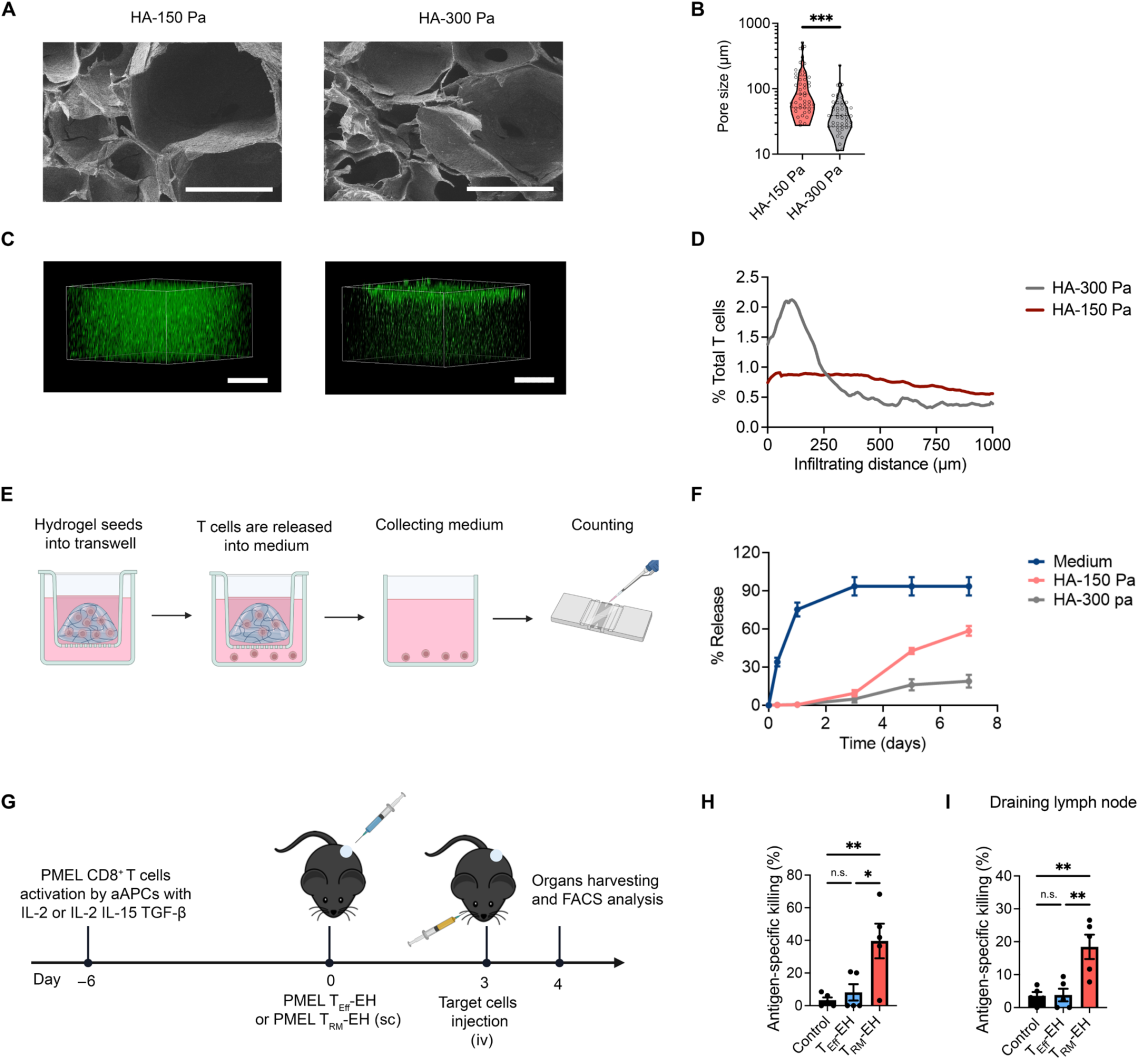
Injectable HA macroporous hydrogel–released T_RM_-like cells display robust antigen-specific cytolytic activity. (**A**) Scanning electron microscopy (SEM) image of HA hydrogel showing macroporous structure throughout the hydrogel; scale bars, 250 μm. (**B**) Pore sizes of HA hydrogel. *n* = 49. Two tailed unpaired Student’s *t* test with Welch’s correction. ***P < 0.001. (**C**) The confocal microscopy three-dimensional (3D) reconstruction images of activated PMEL CD8^+^ T cells migration in different HA hydrogels; scale bars, 500 μm. (**D**) Quantitative analysis of infiltrating distance of CD8^+^ T cells in the HA hydrogel. (**E**) Schematic of the experimental setup to measure T cell release from HA hydrogel. Created with BioRender.com. (**F**) Percentage of cells released from HA hydrogel. (**G**) Schematic of the experimental setup to access antigen-specific killing of adoptively transferred CD8^+^ T cells. sc, subcutaneous; iv, intravenous. PMEL T_RM_-like cells induced in vivo killing in the (**H**) spleens and (**I**) draining lymph nodes. Each bar represents means ± SEM. *n* = 5. One-way ANOVA with Tukey’s multiple comparisons test. *P < 0.05 and **P < 0.01.

We optimized the stiffness and pore size of the hydrogel to maximize T cell migration inside the hydrogel. We used acrylate-grafted HA to cross-link with α, ω-dithiol polyethylene glycol (PEG-SH) to form a hydrogel network under a facile condition at 37°C in phosphate-buffered saline (PBS). We prepared HA hydrogels with two stiffness levels (shear storage modulus *G*′ = 150 and 300 Pa) by modulating the concentrations of HA-Ac and PEG-SH (fig. S7, A and B). The time sweep measurements suggest that the gelation of both 150- and 300-Pa hydrogels occurred quickly within 600 s but required hours to reach the plateau storage modulus. On the basis of these observations, our gelation time was set at 16 hours (fig. S7, C and D) (*39*). Scanning electron microscopy (SEM) images presented macroporous microarchitecture with pore diameters ranging from 27.6 to 508.5 μm and 11.4 to 115.4 μm, respectively (Fig. 3, A and B). The average pore diameters for the two HA hydrogels were 114.6 ± 100.6 μm (*G*′ = 150 Pa) and 44.4 ± 24.4 μm (*G*′ = 300 Pa). We assessed the T cell migration rate in the hydrogel by seeding carboxyfluorescein diacetate succinimidyl ester (CSFE)–labeled CD8^+^ T cells on top of the hydrogel, incubating for 8 hours at 37°C. We imaged the cell-infiltrated hydrogel using confocal laser scanning microscopy. Three-dimensional (3D) reconstruction confocal images revealed that the 150-Pa hydrogel had a 25% higher number of infiltrated cells and a more uniform distribution than the 300-Pa hydrogel (Fig. 3, C and D).

Next, we analyzed the T cell release profile from the 150- and 300-Pa hydrogels. We encapsulated T_RM_-like cells into the 150- and 300-Pa hydrogels and then added them to transwells (pore size, 8 μm) through a 30-gauge needle (fig. S8). We collected the medium in the chamber below at defined time points to determine how many T cells were released into the medium (Fig. 3E). The 150-Pa hydrogel released T_RM_-like cells more quickly over 7 days than the 300-Pa hydrogel (Fig. 3F). On the basis of these results, we used the 150-Pa hydrogel in the following in vivo studies.

To evaluate the antigen-specific cytolytic activity of induced T_RM_-like cells, we first conducted in vitro killing assays using B16-OVA and B16F10 tumor cells. OT-I T_RM_-like cells, which recognize MHC-I–restricted epitopes of chicken OVA, were incubated with B16-OVA cells. PMEL T_RM_-like cells, which recognize GP100 melanoma antigen, were incubated with B16F10 cells. The T_RM_-like cells demonstrated antigen-specific killing capabilities comparable to those of standard effector T cells expanded by nano-aAPCs with IL-2 (T_Eff_ cells) in both tumor cell lines (fig. S9). Given that T_RM_ cells have unique features, such as rapid effector function, enhanced expression of retention markers, and the ability to recruit other immune cells, which likely support T_RM_ in displaying enhanced cytotoxic function in vivo, we performed in vivo killing assays in B6 mice (*40*–*44*) to further assess the function of T_RM_-like cells. On day 0, 1 × 10^6^ PMEL T_RM_-like cells were injected intravenously and compared to the same number of intravenously injected standard PMEL effector T cells. Animals were intravenously injected with 1 × 10^7^ carboxyfluorescein diacetate succinimidyl ester (CFSE)–labeled GP100 peptide–pulsed target cells that can be specifically killed by PMEL T cells and un-pulsed control cells at a 1:1 ratio 3 days after T cell transfer.

Compared to control cells, T_RM_-like cells exhibited robust GP100-specific killing in both the draining lymph nodes and spleens (figs. S10 and S11). In addition, we examined the specific killing of hydrogel-encapsulated PMEL T_RM_-like cells (T_RM_-EH) versus effector T cells (T_Eff_-EH). T_RM_-EH’s in vivo cytolytic activity was significantly higher than T_Eff_-EH in spleens and draining lymph nodes (Fig. 3, G and I, and fig. S12). These findings indicate that T_RM_-like cells exhibited superior systemic cytolytic activity or via hydrogel delivery. Hydrogel-based delivery may further enhance the cytolytic activity of T_RM_-like cells, suggesting that the 150-Pa hydrogel provides a suitable 3D culture environment for T cell encapsulation and survival in vivo.

### T_RM_-like cells delivered with HA hydrogel exhibited better antitumor efficacy than intravenously administered T_RM_-like cells

**Fig. 4.**
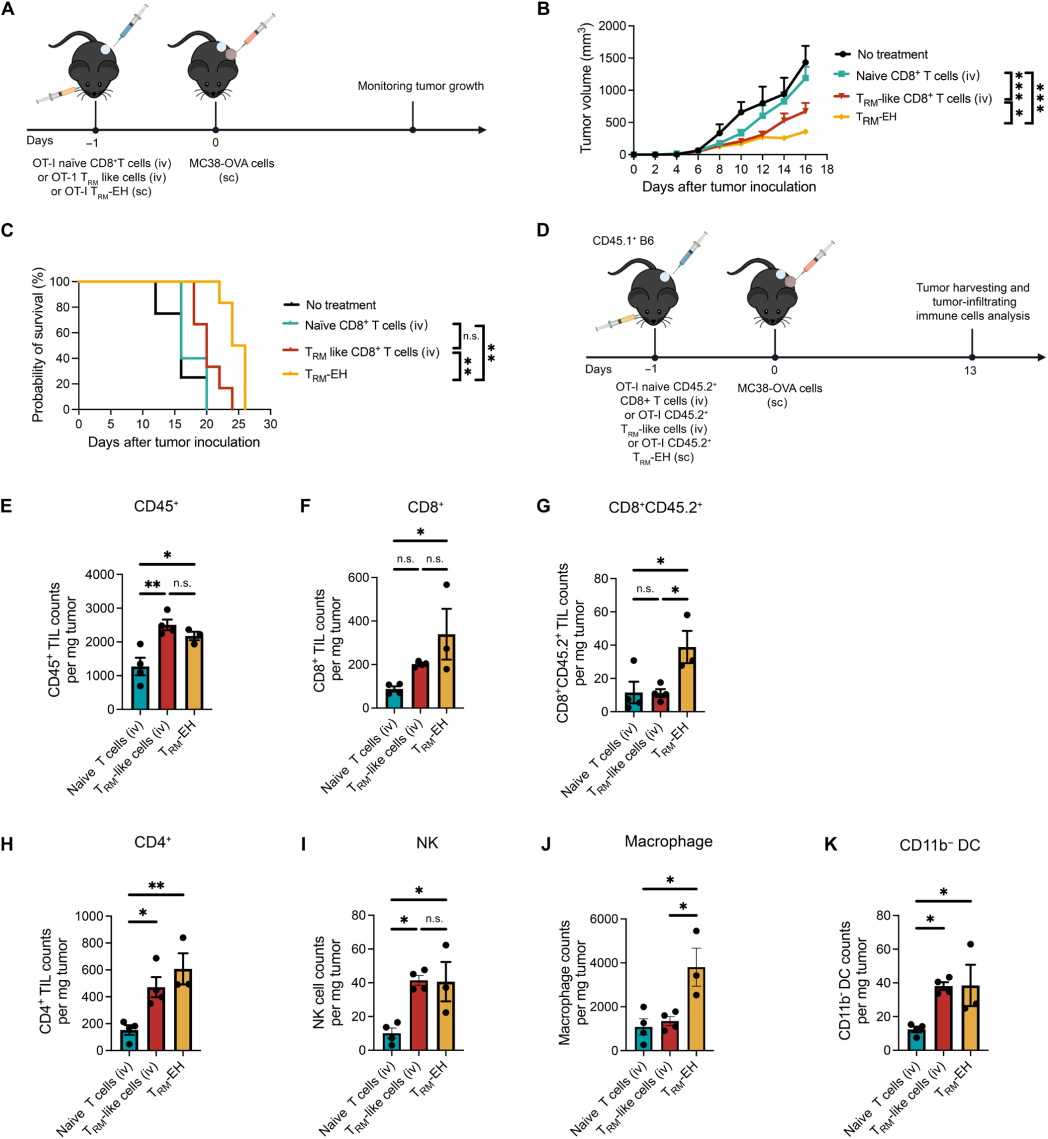
T_RM_-EH exhibits higher antitumor efficacy than intravenously administered T_RM_-like cells. (**A**) Schematic of the experimental design. Mice were administered either naïve OT-I CD8^+^ T cells (intravenous), OT-I T_RM_ CD8^+^ T cells (intravenous), or OT-I T_RM_-EH (subcutaneous) 1 day before subcutaneous MC38-OVA tumor cell injection. (**B**) MC38-OVA tumor growth curves with means ± SEM. *n* = 5 to 6. Two-way ANOVA with Tukey’s multiple comparisons test. *P < 0.05 and ***P < 0.001. (**C**) Mouse survival curve. *n* = 5 to 6. Log-rank test. **P < 0.01. (**D**) Schematic of the experimental design. Mice were administered either naïve OT-I CD8^+^ T cells (intravenous), OT-I T_RM_-like CD8^+^ T cells (intravenous), or OT-I T_RM_-EH (subcutaneous) 1 day before subcutaneous MC38-OVA tumor cell injection. On day 13, tumors were isolated, and tumor-infiltrating immune cells were analyzed. Quantification of total (**E**) CD45^+^, (**F**) CD8^+^, (**G**) CD8^+^ CD45.2^+^, (**H**) CD4^+^, (**I**) natural killer (NK), (**J**) macrophages, and (**K**) CD11b^–^ dendritic cells (DC) in the tumor is shown. Each bar represents means ± SEM. *n* = 3 to 4. One-way ANOVA with Tukey’s multiple comparisons test. *P < 0.05 and **P < 0.01. n.s., not significant.

To assess the in vivo antitumor activity, we compared OT-I T_RM_-EHs (subcutaneous) and naïve OT-I CD8^+^ T cells (intravenous), as well as OT-I T_RM_-like cells (intravenous), in an MC38-OVA tumor prevention model (Fig. 4A). Compared to the mice receiving intravenous naïve CD8^+^ T cells, mice receiving T_RM_-like cells intravenously or T_RM_-EHs significantly delayed tumor formation and progression (Fig. 4B). Moreover, T_RM_-EH showed better antitumor activity than intravenously injected T_RM_-like cells and significantly enhanced survival, highlighting the importance of bypassing homing limitations to improve T_RM_-like cells’ efficacy.

We further analyzed the tumor-infiltrating immune cells in the same MC38-OVA model to mechanistically understand the T_RM_-like cell–mediated antitumor activity. We treated tumor-bearing B6 mice (CD45.1^+^) as described and harvested their tumors on day 13 (Fig. 4D and fig. S13**)**. Both intravenously injected T_RM_-like cells and T_RM_-EH significantly increased the number of CD45^+^ immune cells within the tumor compared to naïve T cells (Fig. 4E). A rise in the total CD8^+^ T cell count was also observed; however, a statistically significant increase occurred only in tumors treated with T_RM_-EHs (Fig. 4F).

To specifically analyze the adoptively transferred CD8^+^ T cells, we used the congenic marker CD45.2. Compared to T_RM_-like cells injected intravenously, hydrogel-released T_RM_-like cells showed increased tumor infiltration (Fig. 4G). In addition, more host-derived CD11b^–^ dendritic cells, natural killer (NK) cells, CD4^+^ T cells, and macrophages were detected in tumors treated with T_RM_-like cells (Fig. 4, H to K). These findings demonstrate that T_RM_-EH can delay tumor generation and progression by enhancing tumor accumulation of exogenous T_RM_-like cells and recruiting an endogenous immune cell population. This aligns with the intrinsic function of T_RM_ cells: secreting pro-inflammatory cytokines and chemokines at a tumor site, increasing adaptive and innate immune cell recruitment (*10*, *14*).

### T_RM_-EH elicited local and systemic immune responses

**Fig. 5.**
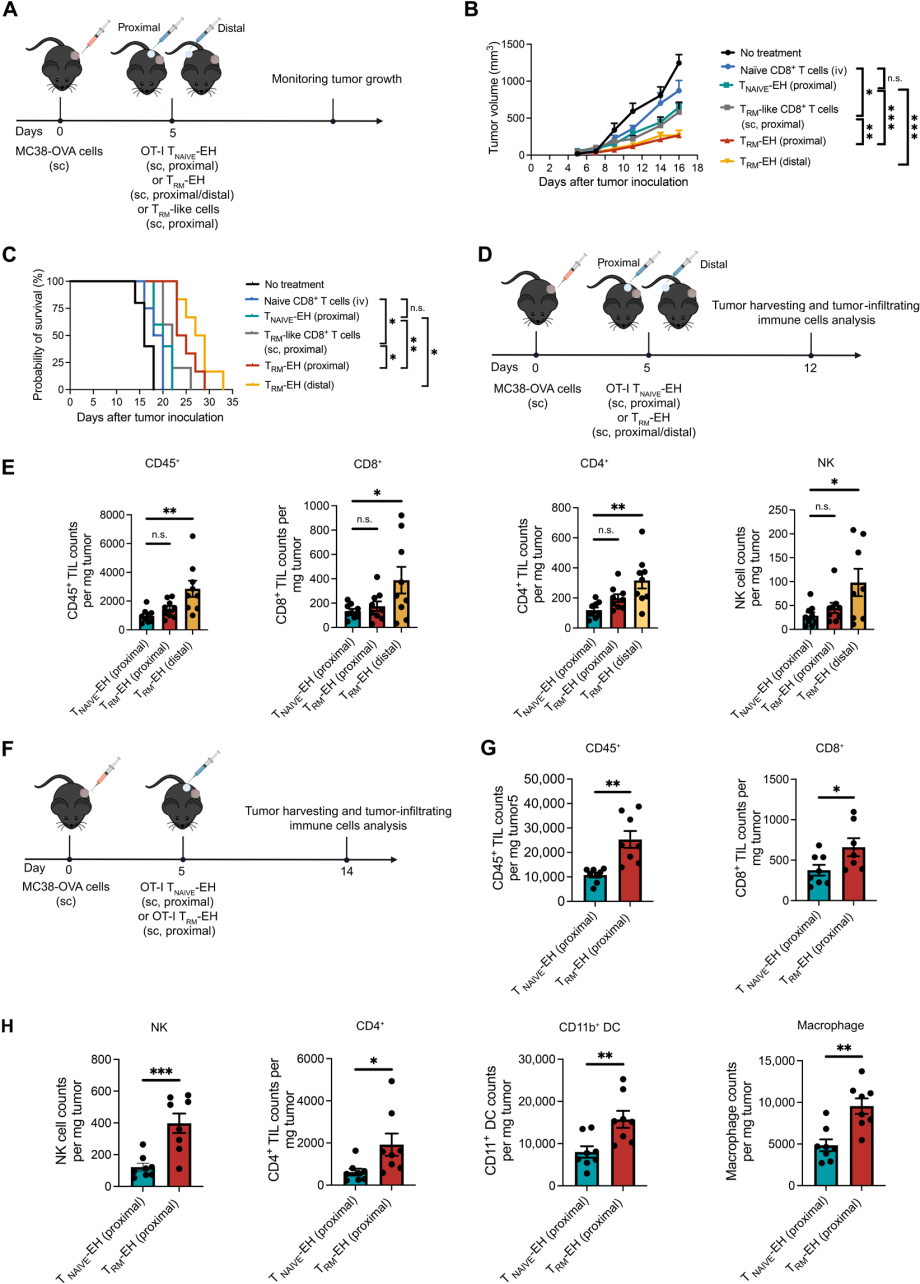
T_RM_-EHs elicited robust local and systemic immune responses in a mouse colon carcinoma model. (**A**) Schematic of the experimental design. OT-I T_RM_-EHs were subcutaneously injected on the same or opposite side of the MC38-OVA tumor 5 days after tumor inoculation. (**B**) MC38-OVA tumor growth curves with means ± SEM. *n* = 5 to 6. Two-way ANOVA with Tukey’s multiple comparisons test. *P < 0.05, **P < 0.01, and ***P < 0.001. (**C**) Mice survival curve. *n* = 5. Log-rank test. *P < 0.05 and **P < 0.01. (**D**) Schematic of the experimental design. OT-I T_RM_-EHs were subcutaneously injected on the same or opposite side of the MC38-OVA tumor 5 days after tumor inoculation. On day 12, tumors were isolated, and tumor-infiltrating immune cells were analyzed. (**E**) Quantification of total CD45^+^, CD8^+^, CD45.2^+^, CD4^+^, and NK cells in the tumor is shown. *n* = 9. One-way ANOVA with Tukey’s multiple comparisons test. *P < 0.05 and ***P < 0.001. (**F**) Schematic of the experimental design. OT-I CD8^+^ T_RM_-EHs were subcutaneously injected on the same side of the MC38-OVA tumor 5 days after tumor cell injection. On day 14, we isolated the tumors and analyzed tumor-infiltrating immune cells. Quantification of total (**G**) CD45^+^, CD8^+^ (**H**) CD4^+^, NK cells, CD11b^+^ dendritic cells, and macrophages in the tumors is shown. Each bar represents means ± SEM. *n* = 8. Two-tailed unpaired Student’s *t* test. *P < 0.05, **P < 0.01, and ***P < 0.001.

We studied the impact of T_RM_-EHs on treatment models. We injected mice subcutaneously with MC-38-OVA or B16-OVA tumor cells. We treated them peritumorally 5 days later, once tumors were palpable, with either naïve OT-I CD8^+^ T cell–encapsulated hydrogels (T_NAIVE_-EHs), OT-I T_RM_-like CD8^+^ T cell suspension (subcutaneous), or OT-I T_RM_-EHs (subcutaneous) (Fig. 5A and fig. S14A). T_RM_-EHs exhibited potent antitumor efficacy and prolonged animal survival compared to treatment with T_NAIVE_-EHs. Hydrogel-mediated release of T_RM_-like cells surpassed the effectiveness of subcutaneously injected T_RM_-like cells, highlighting the advantageous role of hydrogel delivery in augmenting the antitumor impact of these cells. To further assess the efficacy of T_RM_-EHs when implanted at a distant site, we subcutaneously implanted T_RM_-EHs on the tumor’s opposite flank. Treatment with T_RM_-EHs, even at a distal site, significantly inhibited tumor growth, 78 and 53% of MC-38-OVA (Fig. 5B) and B16-OVA (fig. S14B), respectively. Moreover, this treatment significantly improved median survival by 75 and 21% in MC-38-OVA (Fig. 5C) and B16-OVA models, respectively (fig. S14C).

To learn how T_RM_-EHs elicit immune responses in local and distal tumors, we examined tumor-infiltrating immune cells in the MC38-OVA tumor treatment model (Fig. 5, D and F). Notably, T_RM_-EHs, whether implanted locally or at a distant site, increased CD45^+^ immune cell and CD8^+^ T cell counts in the tumors, respectively (Fig. 5, E and G). We also observed higher degrees of infiltration of CD4^+^ T cells and NK cells (Fig. 5, E and H). In addition, T_RM_-EHs markedly increased the number of macrophages and CD11b^+^ DC on day 14 (Fig. 5H). T_RM_-EHs implanted at a distal site had somewhat increased recruitment of macrophage and CD11b^+^ DC cells (fig. S15). These results indicate that T_RM_-EH can serve as potent inducers of antitumor responses locally and in distal tumors.

### T_RM_-EH synergized with PD-1 blockade therapy

**Fig. 6.**
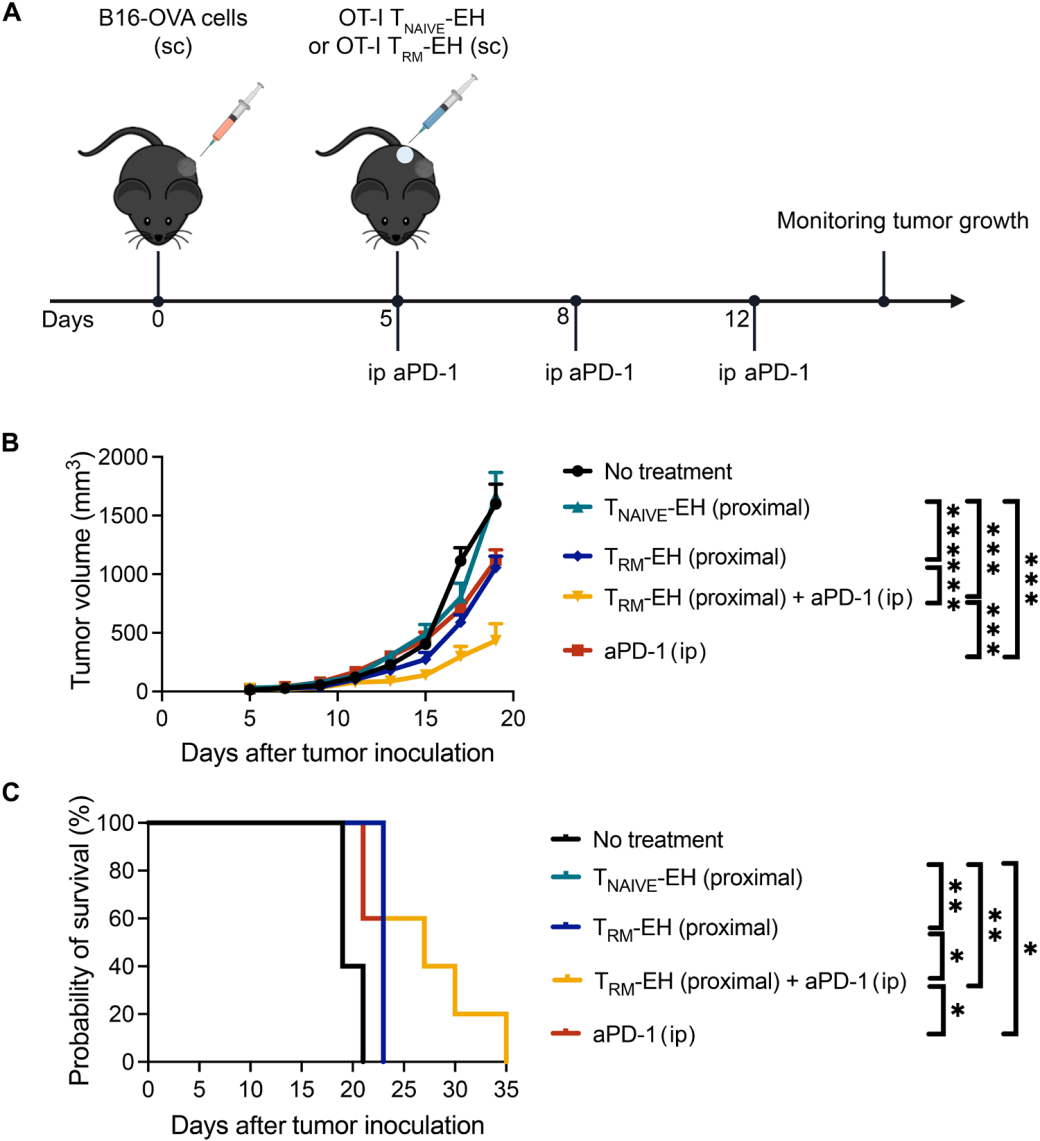
T_RM_-EHs synergized with PD-1 blockade therapy in a mouse melanoma model. (**A**) Schematic of the experimental design. Mice were subcutaneously injected with B16-OVA tumor cells. OT-I T_RM_-EHs were subcutaneously injected 5 days after tumor cell injection. The T_RM_-EHs were injected on the same flank as the tumor, followed by three anti–PD-1 injections on days 5, 8, and 12. (**B**) B16-OVA tumor growth curve with means ± SEM. *n* = 5. Two-way ANOVA with Tukey’s multiple comparisons test. ***P < 0.001. (**C**) Mouse survival curve. *n* = 5. Log-rank test. *P < 0.05 and **P < 0.01. ip, intraperitoneal.

On the basis of the observation that T_RM_-like cells exhibited a partially exhausted phenotype (PD-1^+^ Tim-3^–^) (fig. S16), we examined whether checkpoint blockade inhibitor (CPI) therapy could boost the antitumor activity of T_RM_-like cells. We treated B16-OVA tumor-bearing mice with T_RM_-EHs and anti–PD-1 (Fig. 6A). We observed that combination therapy led to a remarkable suppression of tumor growth by ~73% and exhibited a significant improvement in median survival, extending it by 42%, which is superior to that observed with either treatment with anti–PD-1 or T_RM_-EHs alone (Fig. 6, B and C). Thus, T_RM_-EHs mediate more effective antitumor immunity in the setting of CPI therapy.

### Nano-aAPCs induced functional antigen-specific human CD8^+^ T cells with a T_RM_-like phenotype

**Fig. 7.**
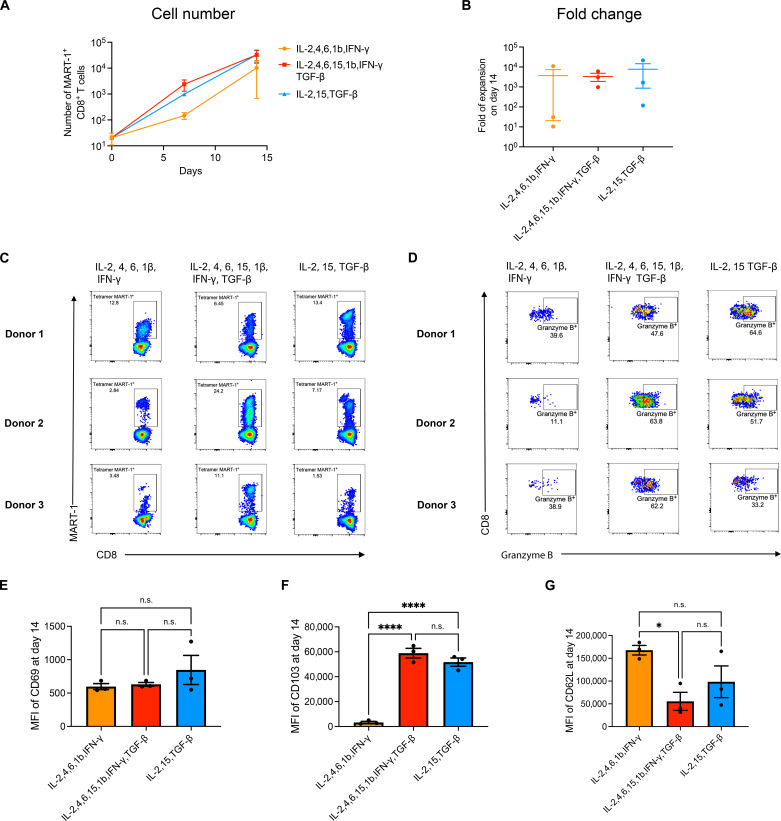
Nano-aAPCs induce functional antigen-specific human CD8^+^ T cells with T_RM_-like phenotype. (**A**) Cell number of MART-1^+^ CD8^+^ T cells expanded by nano-aAPCs in the presence of various cytokine mixes on days 0, 7, and 14. Each bar represents means ± SEM. *n* = 3 donors. (**B**) Fold expansion of MART-1^+^ CD8^+^ T cells expanded by nano-aAPCs in the presence of various cytokine mixes over 14 days. Each bar represents means ± SEM. *n* = 3 donors. (**C**) Tetramer staining of MART-1^+^ CD8^+^ T cells expanded by nano-aAPCs in the presence of various cytokine mixes on day 14 from three healthy donors. (**D**) Granzyme B expression of MART-1^+^ CD8^+^ T cells on day 14. Mean fluorescence intensity of (**E**) CD69 expression, (**F**) CD103 expression, and (**G**) CD62L expression of MART-1^+^ CD8^+^ T cells on day 14. Each bar represents means ± SEM. *n* = 3 donors. One-way ANOVA with Tukey’s multiple comparisons test. *P < 0.05 and ***P < 0.001.

To investigate whether a nano-aAPC platform could be further translated for induction of human T_RM_ cells, human MHC-I molecule human leukocyte antigen (HLA) A2Ig-melanoma antigen recognized by T cells 1 (MART-1) and anti-CD28 were conjugated to nano-aAPCs for expansion of antigen-specific T_RM_ cells from healthy donors’ CD8^+^ T cells in the presence of three different cytokine mixtures. The mixtures were as follows: (i) human T cell expansion cytokine mix, IL-2, IL-4, IL-6, IL-1β, and IFN-γ (*27*); (ii) cytokine mix initially used for mouse T_RM_-like cells generation, IL-2, IL-15, and TGF-β; and (iii) human T_RM_ cytokine mix that combined cytokine mixes 1 and 2, IL-2, IL-4, IL-6, IL-15, IL-1β, IFN-γ, and TGF-β. The hydrodynamic diameter of A2Ig–MART-1 aAPCs was around 140 nm, and each bead was conjugated with 34 ± 18 MHC-I A2Ig–MART-1 molecules (fig. S17). Robust antigen-specific expansion was found on day 14 in both supplemented media with IL-15 and TGF-β from nearly undetectable frequencies and numbers (Fig. 7A and fig. S18) to ~25% of CD8^+^ T cells (Fig. 7C), resulting in up to a 20,000-fold expansion (Fig. 7B). Now, studies of T cell immunity in human tissues have shown that CD69 expression along with CD103 can reliably distinguish human T_RM_ from their circulating counterparts (*45*, *46*). Thus, we used CD69 and CD103 to characterize human T_RM_-like cells in this study. We found that MART-1^+^ CD8^+^ T cells expanded in the media with IL-15 and TGF-β showed predominantly T_RM_-like phenotype (Fig. 7, E to G, and fig. S19). Furthermore, a higher expression of granzyme B was detected in these cells across all three donors compared with MART-1^+^ CD8^+^ T cells expanded without IL-15 and TGF-β after antigen-specific restimulation by A2Ig–MART-1 aAPCs (Fig. 7D). Hence, these results demonstrate that nano-APCs with optimized cytokine milieu show a translational potential for the induction of functional antigen-specific human CD8^+^ T cells with T_RM_-like phenotype from endogenous repertoire.

## DISCUSSION

In recent years, extensive studies have highlighted the important roles of T_RM_ cells in cancer immune surveillance and immunotherapy (*47*). It is now clear that T_RM_ cells contribute to coordinating rapid immune responses against pathogens and cancers (*48*, *49*) and are strongly correlated with improved clinical outcomes in cancers (*15*–*19*). Thus, the adoptive transfer of T_RM_ cells for cancer therapy is highly attractive. Now, research mainly focuses on the adoptive transfer of preprogrammed T cells, such as chimeric antigen receptor T-cell (CAR-T) cells, or other T cell subsets, such as central memory T cells, to promote T_RM_-like cell development in mouse models (*50*–*53*). This development is time-consuming and costly.

This study described a facile nano-aAPC platform to rapidly expand antigen-specific murine and human T_RM_-like cells, yielding large quantities, and achieve effective adoptive T_RM_ transfer by hydrogel delivery. The nano-aAPC–induced CD8^+^ T cells cultured with IL-2, IL-15, and TGF-β proliferated vigorously and had the distinct T_RM_ phenotype CD69^+^CD103^+^CD62L^–^. TGF-β–modulated expression of key T_RM_-associated surface markers (CD103 and CD62L) and core T_RM_-related gene signature confirmed that TGF-β was a central mediator in T_RM_ cell formation and differentiation (*31*). In particular, in the induced murine T_RM_-like cells, the polyfunctionality of CD103^+^ T cells was superior to that of CD103^–^ T cells. On human T_RM_-like cells, a higher frequency of granzyme B^+^ cells in CD103^+^ CD8^+^ T cells was detected across all the donors compared with CD103^−^ CD8^+^ T cells. This finding suggests CD103 may be a vital integrin contributing to T_RM_ residence and potential effector functions (*54*, *55*).

To overcome intrinsic homing limitations following intravenous injection and to maximize the therapeutic potential of nano-aAPC–induced T_RM_-like cells, we optimized a macroporous HA hydrogel to deliver these cells. Compared with the 300-Pa hydrogel, the 150-Pa hydrogel showed higher levels of T cell infiltration and improved the delivery efficiency of nano-aAPC–induced T_RM_-like cells. It showed robust therapeutic efficacy in local and distal tumors by directing antigen-specific tumor-killing and indirect endogenous immune cell recruitment. Further, the combination of T_RM_-like cells and anti–PD-1 exerted a potent synergistic effect in effectively controlling tumor growth in melanoma that was poorly responsive to anti–PD-1 alone. These results highlight the substantial potential of T_RM_-like cells for treating localized and metastatic tumors.

Future studies could explore more cues influencing T_RM_ polarization, such as IL-7 (*28*), IL-12 (*56*), IL-33 (*57*), and CD24 (*58*), using our system. Such studies would enhance understanding of how these signals promote T_RM_ cell stimulation and programming, consequently generating T_RM_ cells with improved protective capacity against pathogens and malignancies. Moreover, the macroporous HA hydrogel provides a suitable T cell infiltration and survival environment. We intend to conduct further research to investigate T_RM_-like cells’ in situ proliferation and differentiation in an enhanced hydrogel system that mimics lymphoid tissue features to enhance T_RM_-based immunotherapy. The induction of patient-derived T_RM_-like cells and the efficient accumulation of those cells within tumor microenvironments while maintaining their robust potency in humanized models are crucial aims for advancing the translation of T_RM_ cell–based immunotherapy in a clinical setting.

In summary, this study provides an effective method for generating antigen-specific T_RM_-like T cells ex vivo. The method uses a nano-aAPC platform and macroporous HA hydrogel to enable the delivery and functions of ex vivo–generated T_RM_-like cells. The hydrogel-delivered T_RM_-like cells showed potent antitumor efficacy alone in locally and distally implanted tumor models and in synergy with anti–PD-1 treatment. The results demonstrate the therapeutic utility of the nano-aAPC–induced T_RM_-like T cells and could improve scientific understanding of T_RM_ cell–mediated antitumor immunotherapy.

## MATERIALS AND METHODS

### Mice

Both PMEL and OT-I transgenic mice were bred and maintained in-house. BALB/c ByJ (CD45.1) and C57BL/6J mice were purchased from the Jackson Laboratory (Bar Harbor, ME, USA). All studies were reviewed and approved by the Institutional Animal Care and Use Committee (IACUC) at Johns Hopkins School of Medicine. The experiments were conducted in accordance with the approved protocol under animal protocol nos. MO23M127 and MO22M385, following the guidelines of the Johns Hopkins IACUC.

### Cells

The MC38-OVA cell line was a gift from Dr. C. Drake (Johns Hopkins University, MD, USA). The B16-OVA cell line was a gift from Dr. J. Powell (Johns Hopkins University, MD, USA). We cultured B16-OVA and MC38-OVA cell lines in RPMI supplemented with l-glutamine, nonessential amino acids, vitamin solution, sodium pyruvate, β-mercaptoethanol, 10% fetal bovine serum, and ciprofloxacin. B16-OVA cells additionally received G418 (400 μg/ml; Gibco, Thermo Fisher Scientific, MA, USA). The human peripheral blood mononuclear cells (PBMCs) were isolated from the blood of healthy volunteers. For the human study, the Johns Hopkins Institutional Review Board (IRB) approved the protocol using human cells, and all healthy volunteers gave written informed consent (Human IRB protocol number: IRB00307779).

### Synthesis and characterization of nano-aAPCs

Kb-immunoglobulin (Ig) and Db-Ig dimers were previously produced and loaded with a specific peptide SIINF (OVA model antigen, SIINFEKL) and GP100 (B16 endogenous melanoma antigen, KVPRNQDWL) in-house as described, respectively (*59*, *60*). A 20-fold molar excess of trans-cyclooctene–PEG4–*N*-hydroxy succinimidyl ester (TCO-PEG4-NHS; Click Chemistry Tools, AZ, USA) was added to Kb-Ig, Db-Ig dimers, or anti-CD28 (BioXcell, clone 37.51, NH, USA) and incubated at room temperature for 1 hour. Then, we transferred the solutions to centrifugal filters (Amicon, molecular weight cutoff of 50 kDa, Millipore, USA) and washed them three times with 500 μl of PBS. Next, 0.7 μmol of tetrazine-PEG5-NHS ester (Tz-PEG5-NHS; Click Chemistry Tools, AZ, USA) was added to 1 mg of 70-nm dextran iron oxide composite particles with amine surface groups (Micromod, Rostock, Germany) and incubated at room temperature for 1 hour. The resulting particles were loaded on the magnetic columns (Miltenyi Biotec, CA, USA) and washed twice with 500 μl of PBS. Subsequently, Tz-labeled iron oxide particles reacted with TCO-labeled pMHC-I dimer (Kb-Ig or Db-Ig dimers) and anti-CD28 at a 5:1 weight ratio at 4°C overnight. Next, we washed the particles twice with 500 μl of PBS and eluted them in an appropriate volume of PBS. The bare nano-aAPCs and protein-conjugated nano-aAPCs were characterized by dynamic light scattering using a Malvern ZetaSizer Pro (Malvern Instruments, Westborough, MA). The nano-aAPCs were diluted at a 1:5 ratio in PBS and sized in a low-volume disposable cuvette using recommended machine settings.

### Determining the protein content on nano-aAPC surfaces

The amount of protein conjugated successfully to the surface of nano-aAPCs was measured using fluorescent antibody detection. The amount of Kb-Ig and Db-Ig was measured by staining with fluorescein isothiocyanate (FITC)–conjugated rat anti-mouse Ig 1, 2, and 3 light chains, clone R26-46 (BD Biosciences, NJ, USA), and the amount of murine anti-CD28 was measured by staining with FITC-conjugated mouse anti-Armenian Syrian hamster IgG, clone G192-1 (BD Biosciences, NJ, USA). The amount of A2-Ig was quantified by staining with FITC-conjugated anti-human HLA-A2, clone BB7.2 (BioLegend, CA, USA), and human anti-CD28 was quantified by FITC anti-mouse IgG2a, clone R19-15 (BD Biosciences, NJ, USA). Protein-conjugated nano-aAPCs were incubated with a 1:100 antibody dilution for 1 hour at 4°C and washed three times, and then fluorescence was detected on a Varioskan LUX multimode microplate reader (Thermo Fisher Scientific, MA, USA). We calculated protein using a fluorescent standard curve of staining antibodies. We determined the number of nano-aAPCs by quantifying the iron content with spectrophotometry. The method for iron quantification is based on the high absorptivity of the orange-red Fe^2+^ with 1,10-phenanthroline complexion (ferrous tris-*o*-phenanthroline) formed at 510 nm. Each sample (20 μl) was transferred to a 1.5-ml Eppendorf tube, and 20 μl of deionized water was prepared as a blank. Then, 20 μl of concentrated hydrochloric acid, 20 μl of 10% hydroxylamine hydrochloride solution, 200 μl of ammonium acetate buffer, 80 μl of 0.2% 1,10-phenanthroline solution, and 860 μl of deionized water were added to each tube. The tubes stood for 20 min to allow for complete complex formation. Then, we measured the absorbance of each sample at 510 nm versus the blank on the Varioskan LUX multimode microplate reader. We used an iron standard curve to calculate the concentration of iron. We calculated the number of nano-aAPCs according to the following equation: Number of nano-aAPCs per milliliter of sample = (6 × 1.31 × iron concentration × 10^9^)/(π × 5.24 g/cm^3^ × radius^3^).

### Antigen-specific T_RM_-like CD8^+^ T cell generation and expansion

For murine antigen-specific T_RM_-like cell generation and expansion, CD8^+^ T cells were isolated from OT-I or PMEL transgenic mouse lymph nodes and spleens using CD8^+^ T cells isolation kits and magnetic columns (Miltenyi Biotec, CA, USA) according to the manufacturer’s protocol. On day 0, isolated CD8^+^ T cells (5 × 10^4^ for each condition) were incubated with nano-aAPCs at a concentration of conjugated Kb-Ig or Db-Ig (18 ng/ml) in the B medium (RPMI supplemented with l-glutamine, nonessential amino acids, vitamin solution, sodium pyruvate, β-mercaptoethanol, 10% fetal bovine serum, and ciprofloxacin), supplemented with either human IL-2 (hIL-2; 5 ng/ml; PeproTech, NJ, USA), or hIL-2 (5 ng/ml) and mouse IL-15 (mIL-15; 100 ng/ml; PeproTech, NJ, USA), or hIL-2 (5 ng/ml), mIL-15 (100 ng/ml), and mouse TGF-β (mTGF-β; 5 ng/ml; BioLegend, CA, USA). We plated the cells in a U-bottom 96-well plate at 5 × 10^4^ cells/ml and 125 μl per well. On days 3 and 5 of culture, cells were subcultured at a split ratio of 1 to 3 and fed with two-third volume of the initial B medium with the same concentration of either hIL-2, hIL-2/mIL-15, or hIL-2/mIL-15/mTGF-β. On day 6, expanded CD8^+^ T cells from each condition were counted and stained with aqua-fluorescent reactive dye (Thermo Fisher Scientific, MA, USA) for 30 min at 4°C to assess for viability. Cells were then stained for 20 min at 4°C in the fluorescence-activated cell sorting (FACS) wash buffer with surface marker antibodies: phycoerythrin (PE)–conjugated CD8a antibody, clone 53-6.7 (BD Pharmingen, NJ, USA); FITC-conjugated CD62L antibody, clone MEL-14 (BioLegend, CA, USA); allophycocyanin (APC)–conjugated CD44 antibody, clone IM7 (BioLegend, CA, USA); polyethylene-cyanine 7 (PE-Cy7)–conjugated CD69 antibody, clone H1.2F3 (BD Pharmingen, NJ, USA); and Brilliant Violet™ (BV421)–conjugated CD103 antibody, clone 2E7 (BioLegend, CA, USA).

For antigen-specific human T_RM_-like cell generation and expansion, CD8^+^ T cells were isolated from healthy volunteers’ PBMCs using CD8^+^ T cells isolation kits and magnetic columns (Miltenyi Biotec, CA, USA) according to the manufacturer’s protocol. On day 0, isolated CD8^+^ T cells (170,000 cells for each condition) were incubated with nano-aAPCs at a concentration of conjugated A2-Ig (200 ng/ml) in the AB medium (RPMI supplemented with l-glutamine, nonessential amino acids, vitamin solution, sodium pyruvate, β-mercaptoethanol, 10% human AB serum, and ciprofloxacin), supplemented with either hIL-2, hIL-4, hIL-6, hIL-1β, and human IFN-γ (hIFN-γ); hIL-2, hIL-4, hIL-6, hIL-15, hIL-1β, hIFN-γ, and human TGF-β (hTGF-β); or hIL-2, hIL-15, and hTGF-β. We plated the cells in a U-bottom 96-well plate at 3.4 × 10^6^ cells/ml and 200 μl per well. On days 3 and 5 of culture, cells were fed with 100 μl of AB medium and twice the concentration of cytokines. On day 7, cells were harvested and counted. Then, the cells were replated at 1.25 × 10^6^ cells/ml with nano-aAPCs at a concentration of conjugated A2-Ig (200 ng/ml) in the AB medium supplemented with different cytokine mixes. On days 10 and 12 of culture, cells were fed with 100 μl of AB medium and twice the concentration of cytokines. On day 14, expanded CD8^+^ T cells from each condition were counted and resuspended in FACS wash buffer containing Human BD Fc Block (BD Biosciences) and 1 μg of MART-1 tetramer or New York esophageal squamous cell carcinoma-1 (NY-ESO-1) tetramers (MBL International Corporation, MA, USA) and allowed to incubate for 30 min at room temperature. After washing, cells were stained with aqua-fluorescent reactive dye (Thermo Fisher Scientific, MA, USA) for 30 min at 4°C to assess viability. Cells were then stained for 20 min at 4°C in the FACS wash buffer with surface marker antibodies: PE-Cy7–conjugated CD3 antibody, clone HIT3a; Brilliant Violet™ 605 (BV605)–conjugated CD45RA antibody, clone HI100; allophycocyanin-cyanine 7 (APC-Cy7)–conjugated CD8a antibody, clone RPA-T8 (BioLegend, CA, USA); BV421-conjugated CD62L antibody, clone DREG56 (BioLegend, CA, USA); peridinin-chlorophyll protein-cyanine 5.5 (PerCP-Cy5.5)–conjugated CD44 antibody, clone IM7 (BioLegend, CA, USA); FITC-conjugated CD69 antibody, clone FN50 (BD Pharmingen, NJ, USA); and APC-conjugated CD103 antibody, clone Ber-ACT8 (BioLegend, CA, USA).

After staining, we washed and resuspended the cells in PBS to perform T_RM_ phenotype analysis on an Attune Flow Cytometer (Thermo Fisher Scientific, MA, USA). FACS data were analyzed by FlowJo v10.8 (Tree Star, OR, USA).

### Nano-aAPC–induced T_RM_-like CD8^+^ T cells in bulk RNA sequencing

On day 6 of culture, OT-I T cells expanded in the medium containing nano-aAPCs in the presence of mIL-2 (T_Eff_) or mIL-2, mIL-15, and mTGF-β (T_RM_-like) were harvested for RNA extraction with a quick-RNA miniprep kit (Zymo research, CA, USA). Then, RNA samples were submitted to Psomagen (MD, USA) to generate RNA sequencing (RNA-seq) libraries using the TruSeq Stranded Total RNA Library Prep Kit (Illumina, CA, USA). Libraries were sequenced by an Illumina NovaSeq X Plus platform.

Demultiplexed fastq files were downloaded and first evaluated with FastQC (0.11.9) (*61*, *62*) and MultiQC (v1.21) (*63*) to confirm read quality. The reads were trimmed by Cutadapt (4.8) (*64*) using parameters: -m 10, -a AGATCGGAAGAGCACACGTCTGAACTCCAGTCA, and -A AGATCGGAAGAGCGTCGTGTAGGGAAAGAGTGT to remove primer reads. Trimmed reads were then imported into R (4.3.2), mapped, and quantified with Rsubread (2.14.2) (*65*) against the mouse reference genome M34 (GRCm39) downloaded from GENCODE (*66*). After generating the feature count matrix, we performed a downstream analysis in DESeq2 (*67*). Genes present in at least three samples were retained, giving a total of 21,475 genes. Log fold change shrinkage was performed with apeglm (*68*) for visualization. The batch effect was modeled and removed with the removeBatchEffect function from limma (3.56.2) (*69*) on transformed and normalized counts calculated with the vst function per recommendations from DESeq2 authors. The batch-corrected reads were used to plot PCA plots and heatmaps. For PCA, the top 1000 genes ordered by *P* value (smallest to largest) were used to calculate the PCs.

DEGs were identified as genes with false discovery rate (FDR) adjusted *P*-value < 0.05 and at least 1.5 × fold change (log_2_ fold change > 0.58). We performed overrepresentation analysis (ORA) analysis with clusterProfiler (4.8.3) (*70*) on gene sets from the GO biological process. Up-regulated DEGs (log_2_ fold change > 0) and down-regulated DEGs (log_2_ fold change < 0) were passed into the enrichGO function with *q*-value cutoff of 0.05 and all the genes in our dataset as the universe. Redundant terms were then reduced with the simplify function from GOSemSim (*71*). We then plotted up-regulated and down-regulated pathways from ORA, respectively. GO GSEA was performed on all genes with average normalized counts > 10 (baseMean > 10) using the gseGO function.

### Nano-aAPC–induced T_RM_-like CD8^+^ T cell functionality analysis

For the murine T_RM_-like CD8^+^ T cells, on day 6, PMEL and OT-I T cells expanded in the medium containing nano-aAPCs in the presence of IL-2, IL-15, and mTGF-β (T_RM_-like cells) were restimulated with PMA/ionomycin (1:500) (BioLegend, CA, USA) and brefeldin A (1:1000) (BD Biosciences Pharmingen, NJ, USA) for 6 hours at 37°C, 5% CO_2_. We used unstimulated T_RM_-like cells as a negative control. After incubation for 6 hours, the treated cells were washed in PBS and stained with aqua-fluorescent reactive dye (Thermo Fisher Scientific, MA, USA) for 30 min for dead cell exclusion. We then added PerCy-Cy5.5–conjugated CD8 antibodies, clone 53-6.7 (BioLegend, CA, USA); BV421-conjugated CD103 antibodies, clone 2E7 (BioLegend, CA, USA); and PE-Cy7–conjugated CD69 antibodies, clone H1.2F3 (BD Pharmingen, NJ, USA) for surface marker staining. The stained cells were washed, fixed, and permeabilized by a BD Cytofix/Cytoperm Fixation/Permeabilization kit (BD Biosciences, NJ, USA) according to the manufacturer’s protocol. Subsequently, T_RM_-like cells were washed and stained intracellularly with APC-Cy7–conjugated IFN-γ antibodies, clone XMG1.2 (BioLegend, CA, USA); PE-conjugated TNF-α antibodies, clone MP6-XT22 (BioLegend, CA, USA); and FITC-conjugated IL-2 antibodies, clone JES6-5H4 (BioLegend, CA, USA) for 30 min at 4°C in the FACS wash buffer. Cells were washed and resuspended in PBS for flow cytometric analysis on an Attune Flow Cytometer (Thermo Fisher Scientific, MA, USA). The data were analyzed by FlowJo v10.8 (Tree Star, OR, USA).

For the human T_RM_-like CD8^+^ T cells, on day 14, cells expanded by nano-aAPCs with different cytokine mixes were restimulated with A2Ig–MART-1 aAPCs and Golgi plug for 6 hours and then stained with MART-1 tetramers or NY-ESO-1 tetramers (MBL, MA, USA). Cells were then stained for 20 min at 4°C in the FACS wash buffer with surface marker antibodies: APC-Cy7–conjugated CD8a antibody, clone RPA-T8 (BioLegend, CA, USA); FITC-conjugated CD69 antibody, clone FN50 (BD Pharmingen, NJ, USA); and APC-conjugated CD103 antibody, clone Ber-ACT8 (BioLegend, CA, USA). Cells were subsequently fixed and permeabilized using a BD Cytofix/Cytoperm Fixation/Permeabilization kit and stained with Pacific Blue–conjugated granzyme B antibodies, clone GB11 (BioLegend, CA, USA) at 4°C for 30 min. Cells were washed and resuspended in PBS for flow cytometric analysis as previously described.

### Antigen-specific in vitro killing assay

We generated OT-I or PMEL CD8^+^ T_RM_-like cells or T_Eff_ cells by incubating isolated CD8^+^ T cells with nano-aAPC and IL-2, IL-15, and TGF-β or IL-2 only. B16F10 and B16-OVA cancer cells were used as target cells. A cell suspension (100 μl; 5000 cells per well) of either B16F10 or B16-OVA cell suspension was seeded in a 96-well plate. Stimulated PMEL or OT-I T cells (100 μl) were then added to each well of the plate at varying effector-to-target cell ratios: 0.01, 0.1, 1, 10, and 50. The plates were incubated overnight in a CO_2_ incubator. After incubation, the T cells were carefully removed. Next, 100 μl of 10% cell counting kit-8 (CCK-8) solution (Abcam, Cambridge, UK) was added to each well of the plate. The plate was then incubated for 1 to 4 hours. The absorbance at 450 nm was measured on Varioskan LUX multimode microplate reader.

### Antigen-specific in vivo killing assay

As described above, we generated PMEL Thy1.1^+^ CD8^+^ T_RM_-like cells by incubating isolated CD8^+^ T cells with nano-aAPC and IL-2, IL-15, and TGF-β. On day 0, either PMEL Thy1.1^+^ CD8^+^ T_RM_-like cells or PMEL Thy1.1^+^ CD8^+^ T cells expanded by nano-aAPCs together with IL-2 (T_Eff_ cells) were adoptively transferred retro-orbitally, at 1 × 10^6^ cells per Thy1.2 B6 mouse. For other treatment groups, we implanted 100 μl of hydrogel containing 1 × 10^6^ PMEL Thy1.1^+^ CD8^+^ T_RM_-like cells (T_RM_-EH) or 1 × 10^6^ PMEL Thy1.1^+^ CD8^+^ effector T cells (T_Eff_-EH) into the right flank of Thy1.2 B6 mice. We used B6 mice receiving no treatment as negative controls. On day 3, every 4 × 10^7^ isolated Thy1.2^+^ B6 splenocytes were differently labeled with high (0.5 μM) and low (0.033 μM) levels of CFSE in 1 ml of PBS. Then, we pulsed the splenocytes labeled with high levels of CFSE with GP 100 peptide, which PMEL CD8^+^ T cells can recognize. We mixed the peptide-pulsed CFSE-high splenocytes and un-pulsed CFSE-low splenocytes at a 1:1 ratio. We injected 1 × 10^7^ mixed splenocytes retro-orbitally into recipient Thy1.2^+^ B6 mice in 100-μl volume. After 1 day, we euthanized the mice. Their inguinal lymph nodes, axillary lymph nodes, and spleens were harvested and minced. The resulting cell suspensions were lysed with the ammonium-chloride-potassium (ACK) lysing buffer and filtered through 70-μm cell strainers. Single-cell suspensions were washed and resuspended in PBS. Cells were stained with LIVE/DEAD Fixable Aqua to exclude dead cells. We then stained the cells with PE-conjugated CD8 antibodies, clone 53-6.7 (BD Pharmingen, NJ, USA); PerCP-conjugated CD90.1 (Thy1.1) antibodies, clone OX-7 (BioLegend, CA, USA); and APC-conjugated CD90.2 (Thy1.2) antibodies, clone 53-2.1 (BD Pharmingen, NJ, USA) in the FACS wash buffer for 20 min at 4°C. Cells were washed and resuspended in PBS for flow cytometric analysis to determine the presence of CFSE-labeled cells.

Experiments were performed in quadruplicate. We calculated the percentage of antigen-specific killing activity according to the following equation: Ratio = non-pulsed control T cell percentage: peptide-pulsed target T cell percentage (CFSE-L peak:CFSE-H peak), % specific killing = [1 − (experimental ratio/non-transferred control ratio)] × 100.

### HA hydrogel preparation

We prepared HA hydrogel according to the protocol in previous studies (*35*). We added glycidyl acrylate (TCI America Inc., Portland, OR, USA) to an HA solution, which we prepared by dissolving sodium hyaluronate with a molecular weight of 1.5 × 10^6^ Da (research grade, LifeCore Biomedical Inc., Chaska, MN, USA) in PBS for acrylate modification. The obtained acrylated HA (HA-Ac) was then reacted with dithiol poly (ethylene glycol) (PEG-SH, JenKem Technology, Plano, TX, USA). HA hydrogel at stiffness levels of 150 and 300 Pa was prepared at HA concentrations of 7.5 and 10 mg/ml, respectively, when PEG-SH concentrations were 4.32 and 5.75 mg/ml, correspondingly. We transferred 200 μl of the mixed precursor hydrogel solution into each well of a 48-well plate and placed it at 37°C for 16 hours for gelation. To characterize the mechanical properties of the HA hydrogel. The amplitude sweep and frequency sweep of the hydrogel (shear storage modulus *G*′ and loss modulus *G″*) were measured with an ARG2 rheometer (TA Instruments, DE, USA). The injectability of the 150-/300-Pa HA hydrogel was measured using INSTRON 34SC-05 (Instron, Norwood, MA, USA) with 27-gauge × 0.5 inches (1.27 cm) SERiJECT needle (TSK Laboratory, Japan) and flow speed of 1 ml/min (*72*) (*n* = 3, sample size = 0.5 ml per test, according to the common R&D industrial practice). The HA hydrogel gelation process was measured in a 2-hour period in situ using an ARG2 rheometer (TA Instruments, DE, USA). The initial mixture volume was 0.5 ml and placed at 37°C for gelation while measuring rheology continuously. During the experiment, the compartment was covered to avoid evaporation of the forming hydrogel to maintain constant sample volume during the 2-hour measurement. We observed the inner porosity of HA hydrogel at 150 and 300 Pa using an SEM (Thermo Fisher Scientific Helio G4 UC, MA, USA). We freeze-dried gelated HA hydrogels for 24 hours and then imaged cross sections of the HA hydrogels at an accelerating voltage of 5 kV.

### T cell infiltration into HA hydrogel

We prepared HA hydrogels at stiffness levels of 150 and 300 Pa, as described above. Then, we loaded CFSE-labeled CD8^+^ T cells into the hydrogel at 10 million cells/ml and incubated them overnight at 37°C. We imaged cell infiltration from top to bottom of the hydrogel using a Zeiss LSM 510 Meta Confocal microscope (Zeiss, Germany).

### T cells released from HA hydrogel

We prepared HA hydrogels at stiffness levels of 150 and 300 Pa, as described above. Then, we loaded ex vivo–generated OT-I T_RM_-like cells into the hydrogel at 1 × 10^7^ cells/ml of hydrogel and incubated them for 3 to 4 hours at 37°C until the hydrogel completely absorbed the medium. We injected 200 μl of hydrogel into a transwell insert with a 40-gauge needle. We added 1 ml of fresh B medium supplemented with IL-2 to the bottom chamber and placed the plate in the 5% CO_2_ incubator at 37°C with 200 rpm shaking. At definite time points, we counted cells in the bottom chamber and moved the insert with the hydrogel to another well containing fresh B medium.

### Tumor-preventive effect of T_RM_-like CD8^+^ T cells in MC38-OVA colon carcinoma

We generated OT-I T_RM_-like CD8^+^ cells by incubating OT-I CD8^+^ T cells with cognate pMHC-I conjugated nano-aAPCs in a medium containing IL-2, IL-15, and TGF-β for 6 days, as previously described. We transferred OT-I T_RM_-like cells retro-orbitally at 2 × 10^6^ cells per mouse. We used mice treated with retro-orbitally injected naïve OT-I CD8^+^ T cells or without any cells as negative controls. In addition, we seeded HA hydrogel with OT-I T_RM_-like cells at 1 × 10^7^ cells/ml and incubated it for 3 to 4 hours at 37°C until the hydrogel completely absorbed the medium. We implanted 200 μl of hydrogel subcutaneously on the right flank of mice on the same day. After 1 day, 5 × 10^5^ MC38-OVA cells were inoculated subcutaneously on the right flank of each C57BL/6 mouse. We monitored the tumors starting 2 days after tumor inoculation. Mice were euthanized when tumor volume reached 2000 mm^3^. We calculated tumor volumes using the following equation: *V* = *D* × *d*^2^/2, where *V* is volume (cubic millimeters), *D* is the larger diameter (millimeters), and *d* is the smaller diameter (millimeters).

### Tumor-infiltrating analysis in the MC38-OVA prevention model

Mice were treated with either 2 × 10^6^ naïve OT-I CD8^+^ T cells (intravenous), 2 × 10^6^ OT-I T_RM_-like cells (intravenous), or 200 μl of hydrogel containing 2 × 10^6^ OT-I T_RM_-like cells (subcutaneous) 1 day before MC38-OVA cell inoculation and euthanized 13 days later. Tumor cell suspensions were prepared from solid tumors by enzymatic digestion in B medium containing collagenase (1 mg/ml; Sigma-Aldrich, USA), deoxyribonuclease I (20 U/ml; Sigma-Aldrich, USA), and hyaluronidase (0.1 mg/ml; Sigma-Aldrich, USA) with constant stirring in the 5% CO_2_ incubator at 37°C for 50 min. The resulting tumor was minced, passed through a 70-μm cell strainer, and washed twice with B medium. Subsequently, cell suspensions were resuspended in 10 ml of B medium. An equal volume of mouse-lymphocyte cell separation medium (Cedarlane, Southern Ontario, Canada) was added later. The cells were centrifuged for 20 min at 3000 rpm with the brake off. Then, the cells at the interface were collected and washed with B medium. The cells were seeded in T125 flasks and placed in the 37°C incubator overnight. Then, the non-adherent T cells were harvested and labeled with anti-CD90 microbeads according to the manufacturer’s instructions (Miltenyi Biotec, CA, USA) and purified using magnetic columns. After purification, we analyzed single-cell suspensions using an Attune Flow Cytometer (Thermo Fisher Scientific, MA, USA).

We used antibodies against the following surface markers in the analysis: APC-conjugated anti-CD45, clone 30-F11 (BioLegend, CA, USA); BV605-conjugated anti-CD45.1, clone A20 (BioLegend, CA, USA); PE-conjugated anti-CD45.2, clone 104 (BioLegend, CA, USA); FITC-conjugated anti-CD44, clone IM7 (BioLegend, CA, USA); Alexa Fluor 700–conjugated anti-CD62L, clone MEL-14 (BioLegend, CA, USA); PE-Cy7–conjugated anti-CD69, clone H1.2F3 (BD Pharmingen, NJ, USA); BV421-conjugated anti-CD103, clone 2E7 (BioLegend, CA, USA); PE-conjugated anti-CD3, clone 17A2 (BioLegend, CA, USA); PerCP-Cy5.5–conjugated anti-CD11c, clone N418 (BioLegend, CA, USA); APC-R700–conjugated anti-CD11b, clone M1/70 (BioLegend, CA, USA); BV421-conjugated anti-Ly6c, clone HK1.4 (BioLegend, CA, USA); PE-Dazzle 594-conjugated anti-Ly6g, clone 1A8 (BioLegend, CA, USA); FITC-conjugated anti–IA-IE, clone M5/114.15.2 (BioLegend, CA, USA); Alexa Fluor 647–conjugated anti-CD335, clone 29A1.4 (BioLegend, CA, USA); and PE-Cy7–conjugated anti-CD19, clone 6D5 (BioLegend, CA, USA). The data were analyzed using FlowJo v10.8 (Tree Star, OR, USA).

### Tumor inhibitory effect of T_RM_-like CD8^+^ T cells in MC38-OVA colon carcinomas

OVA-specific naïve and T_RM_-like cells were loaded into hydrogel as previously described. MC38-OVA cells (3 × 10^5^) were inoculated subcutaneously on the right flank of each C57BL/6 mouse. After 5 days, mice were injected subcutaneously with either 2 × 10^6^ T_RM_-like cells suspended in PBS on the same flank of the tumor or 200 μl of hydrogel seeded with 2 × 10^6^ T_RM_-like cells on the same flank or opposite flank of the tumor. We used mice administered with naïve CD8^+^ T cells loaded hydrogel on the same flank of the tumor or without any treatments as negative controls. Tumors were measured with a digital caliper starting 5 days after tumor inoculation. Mice were euthanized when tumor volume reached 2000 mm^3^. We calculated tumor volumes using the following equation: *V* = *D* × *d*^2^/2, where *V* is volume (cubic millimeters), *D* is the larger diameter (millimeters), and *d* is the smaller diameter (millimeters).

### Tumor-infiltrating analysis in the MC38-OVA treatment model

Mice were treated subcutaneously with 200 μl of hydrogel seeded with 2 × 10^6^ OT-I T_RM_-like CD8^+^ T cells on the same or opposite flank of tumor 5 days after 3 × 10^5^ MC38-OVA tumor cells inoculation. Mice were injected subcutaneously with 200 μl of hydrogel containing 2 × 10^6^ naïve OT-I CD8^+^ cells on the same flank of the tumor as negative controls. We euthanized the mice 7 days later and harvested and processed the tumors as described above. In addition, mice were treated subcutaneously with 200 μl of hydrogel seeded with 2 × 10^6^ OT-I T_RM_-like CD8 T cells or naïve OT-I CD8^+^ cells on the same flank of the tumor 5 days after 3 × 10^5^ MC38-OVA tumor cells inoculation. After 9 days, tumors were isolated and processed as described previously for flow cytometry analysis. Single-cell suspensions were analyzed using an Attune Flow Cytometer (Thermo Fisher Scientific, MA, USA), and the antibodies used in the analysis were as previously described. The data were analyzed using FlowJo v10.8 (Tree Star, OR, USA).

### Tumor-inhibitory effect of T_RM_-like CD8^+^ T cells in B16-OVA melanomas

B16-OVA cells (2 × 10^5^) were inoculated subcutaneously on the right flank of each C57BL/6 mouse. After 5 days, mice were subcutaneously implanted with 200 μl of hydrogel containing 2 × 10^6^ T_RM_-like cells in combination with 200 μg of anti–PD-1 monoclonal antibody administered via intraperitoneal injection on days 5, 8, and 12. We administered anti–PD-1 antibody therapy alone and OVA-specific T_RM_-like cell–loaded hydrogels as a control. In addition, we implanted OVA-specific T_RM_-like cell–loaded hydrogel on the opposite flank of the tumor in the B16-OVA model. We measured tumors with a digital caliper starting 5 days after tumor inoculation. Mice were euthanized when tumor volume reached 2000 mm^3^. We calculated tumor volumes using the following equation: *V* = *D* × *d*^2^/2, where *V* is volume (cubic millimeters), *D* is the larger diameter (millimeters), and *d* is the smaller diameter (millimeters).

### Statistical analysis

Data were analyzed using the statistical software program Prism 9 (GraphPad Software Inc., La Jolla, CA) and are reported as means (± SEM). Student’s *t* test assessed differences between two groups, and differences among multiple groups were assessed by one- and two-way analyses of variance (ANOVAs) with Tukey’s multiple comparisons test. We used repeated measure two-way ANOVAs with Tukey’s multiple comparisons tests to compare tumor growth curves, and log-rank tests were used to compare survival curves. Differences of *P* < 0.05 were considered statistically significant.

## Supplementary Material

20241213-1
