# Supplementary material for: Ex vivo expansion and hydrogel-mediated in vivo delivery of tissue-resident memory T cells for immunotherapy
Source: Sci Adv. 2024 Dec 13;10(50):eadm7928. doi: 10.1126/sciadv.adm7928 (PMC11641059; doi:10.1126/sciadv.adm7928)
Supplement: Supplementary file 2 — Figs. S1 to S19 [file sciadv.adm7928_sm.pdf]

Science Advances
AAAS

Supplementary Materials for

Ex vivo expansion and hydrogel-mediated in vivo delivery of tissue-resident memory T cells for immunotherapy

Shuyi Li *et al.*

Corresponding author: Hai-Quan Mao, hmao@jhu.edu; Jonathan P. Schneck, jschnec1@jhmi.edu

Sci. Adv. **10**, eadm7928 (2024)
DOI: 10.1126/sciadv.adm7928

This PDF file includes:

Figs. S1 to S19

Supplementary Figures:

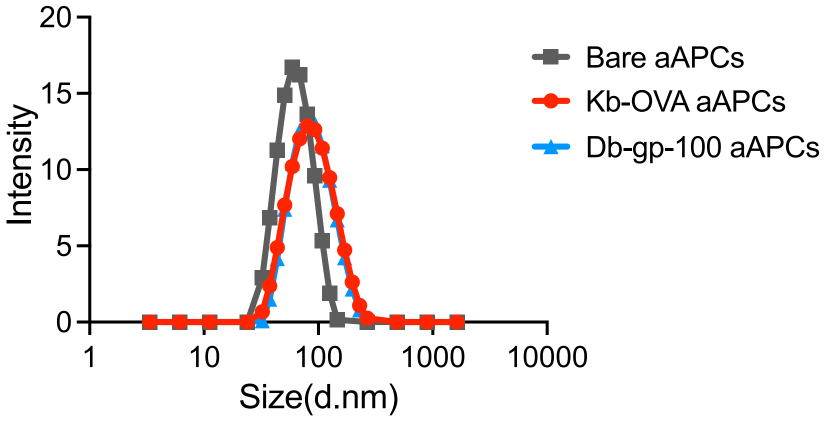

Figure S1. Size distribution of nano-aAPCs before and after protein conjugation.

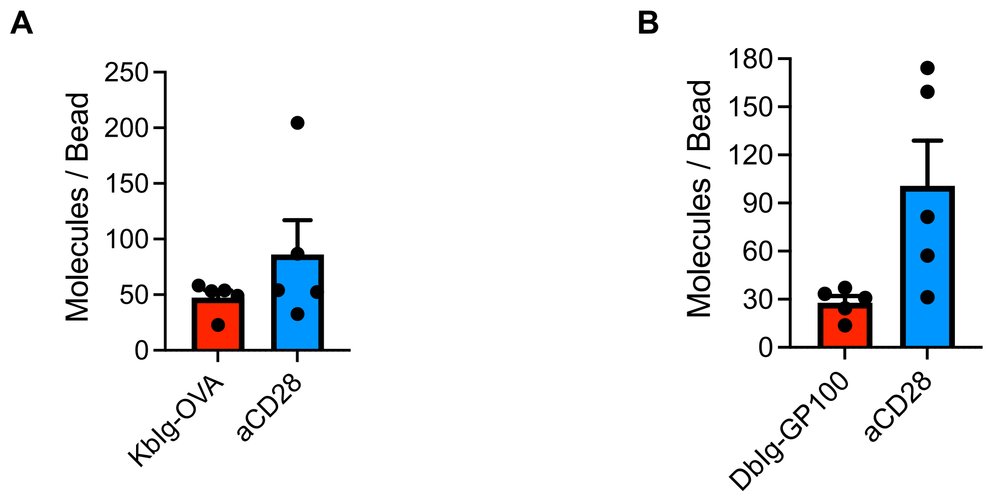

Figure S2. Quantification of KbIg-OVA or DbIg-GP100 and aCD28 conjugated to aAPCs. **(A)** KbIg-OVA/aCD28 Beads **(B)** DbIg-GP100/aCD28 Beads. Each bar represents mean ± s.e.m.. *n* = 5.

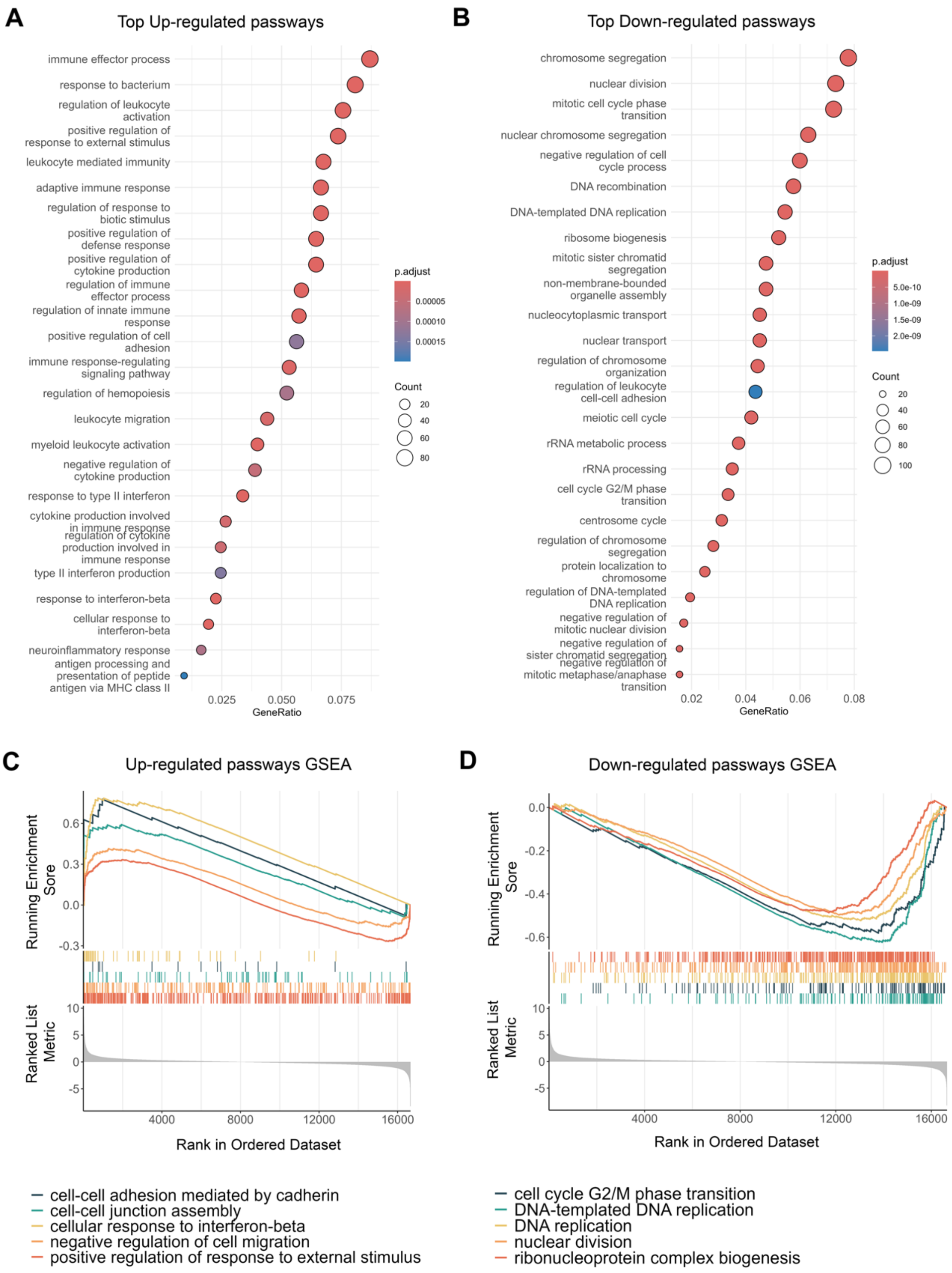

Figure S3. GO enrichment analysis and GSEA of differentially expressed (DE) genes between OT-I CD8^{+} T cells expanded by aAPCs in the presence of IL-2, IL-15 and TGF-β (T$_{RM}$-like) or IL-2 (T$_{Eff}$)

(A) up-regulated gene set and **(B)** down-regulated gene set in T$_{RM}$-like cells **(C)** five GSEA up-regulated pathways and (**D**) five GSEA down-regulated pathways in T$_{RM}$-like cells

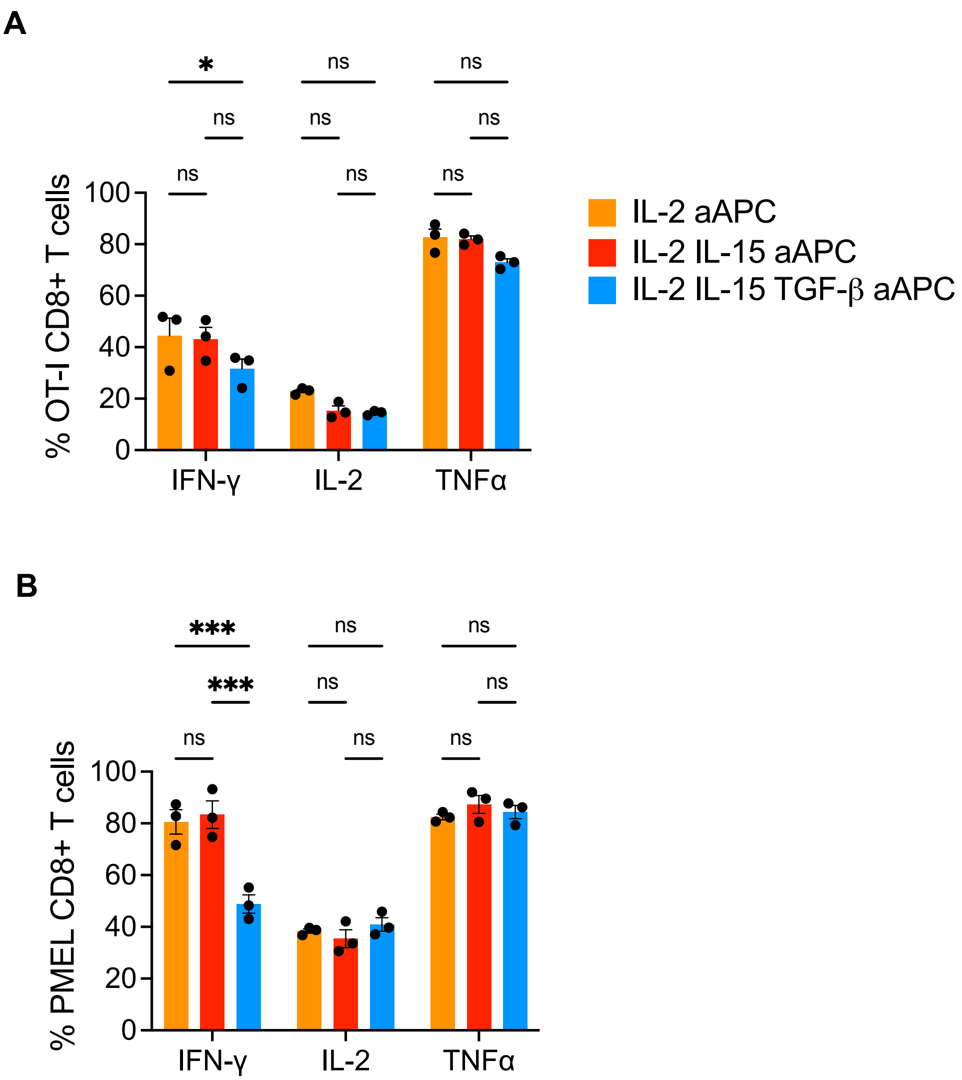

Figure S4. Cytokines production of nano-aAPC-induced CD8$^+$ T cells with different cytokines mixes

(A) OT-I **(B)** PMEL. Each bar represents mean ± s.e.m.. $n = 3$. Two-way ANOVA with Tukey's multiple-comparisons test. *$p < 0.05$, ***$p < 0.001$.

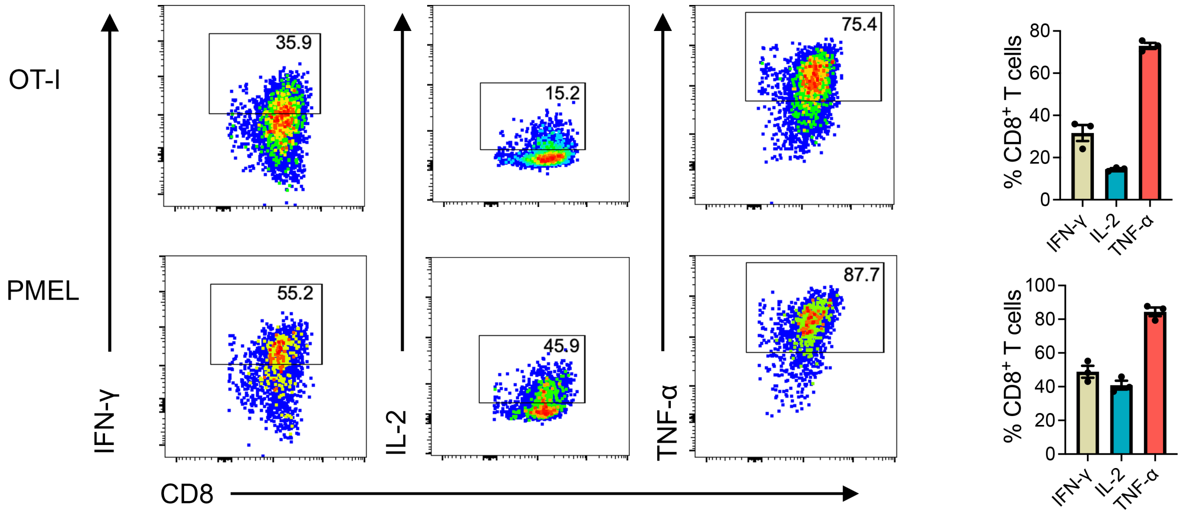

Figure S5 Day 6 cytokine production of PMEL and OT-I CD8$^+$ T cells expanded in the medium containing nano-aAPCs, IL-2, IL-15, and TGF-β. Each bar represents mean ± s.e.m.. *n* = 3.

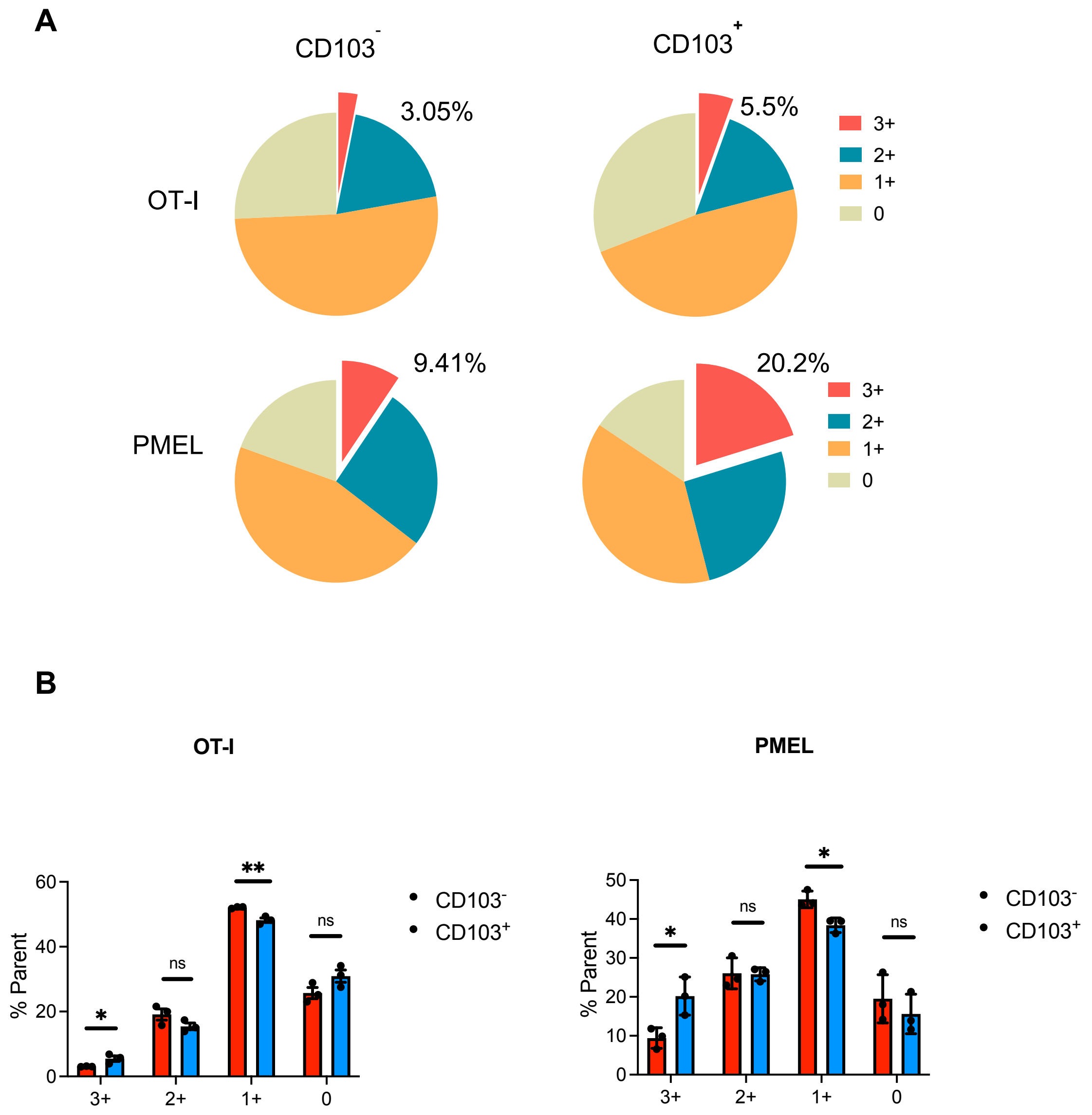

Figure S6. Polyfunctionality of nano-aAPC-induced T_{RM} subsets with varying expression of CD103 markers.
(A) OT-I Pie charts showing the polyfunctionality profile of $CD8^{+}CD103^{+}$ or $CD8^{+}CD103^{-}$ T-cells at Day 6. IL-2, IFN-γ, and TNF-α single (1+), double (2+), or triple positive (3+) cells were assessed. **(B)** PMEL, Bar charts represent the proportion of $CD103^{-}$ or $CD103^{+}$ $CD8^{+}$ T-cells secreting none or any (1, 2, or 3) of the three cytokines IFN-γ, TNF-α, and IL-2. Each bar represents mean ± s.e.m.. $n = 3$. Two tailed unpaired Student's t-test. *$p < 0.05$, **$p < 0.01$.

A

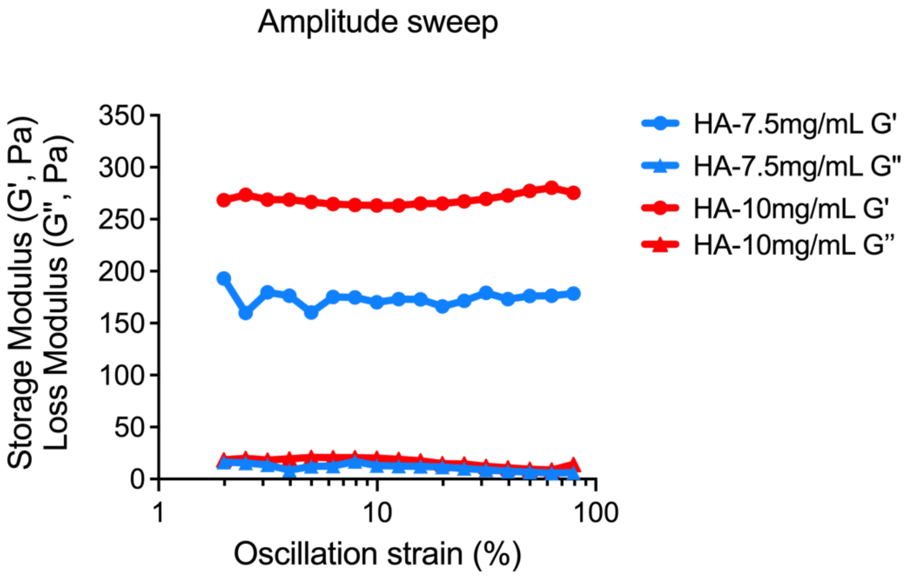
Amplitude sweep
Storage Modulus (G', Pa)
Loss Modulus (G'', Pa)
350
300
250
200
150
100
50
0
1
10
100
Oscillation strain (%)
HA-7.5mg/mL G'
HA-7.5mg/mL G''
HA-10mg/mL G'
HA-10mg/mL G''

B

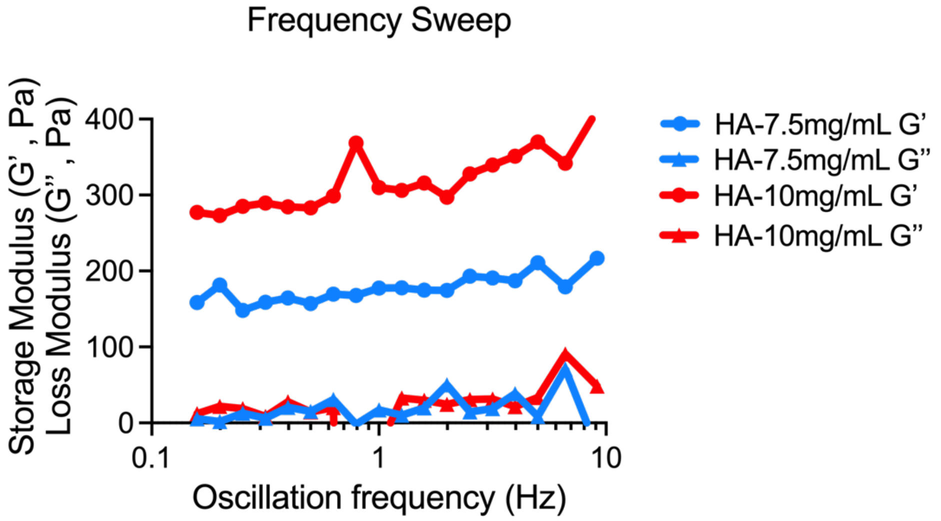
Frequency Sweep
Storage Modulus (G' , Pa)
Loss Modulus (G'' , Pa)
400
300
200
100
0
0.1
1
10
Oscillation frequency (Hz)
HA-7.5mg/mL G'
HA-7.5mg/mL G''
HA-10mg/mL G'
HA-10mg/mL G''

C

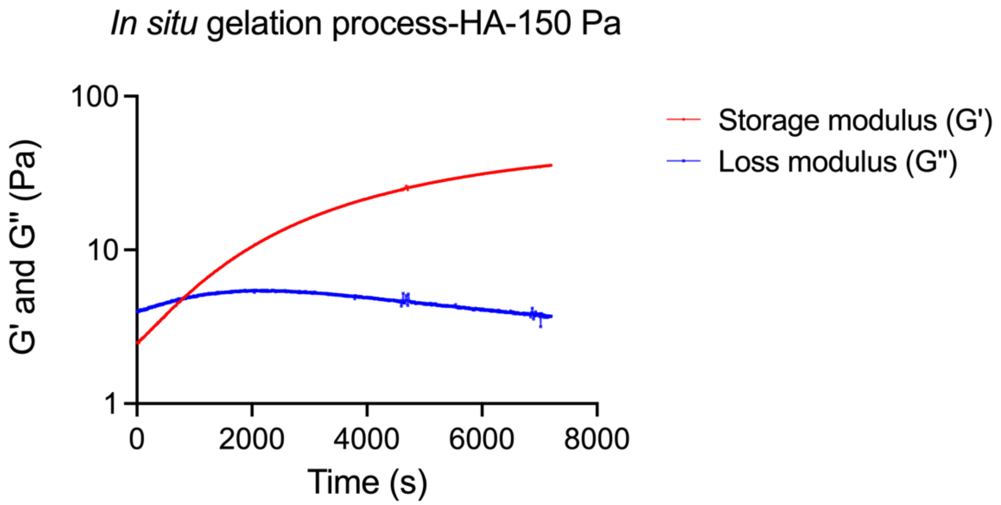

D

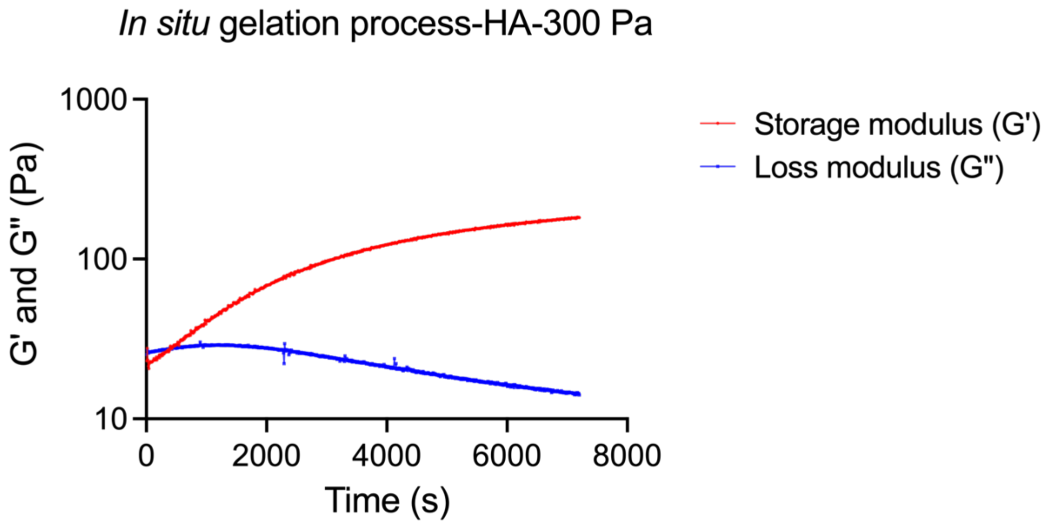

Figure S7. Rheological properties

(A) oscillation amplitude sweep of HA hydrogel (storage modulus G' and loss modulus G" as a function of oscillatory stress) of HA hydrogel. **(B)** Storage modulus G' and loss modulus (G") of the hydrogel as a function of frequency. **(C)** Gelation kinetics of the 150-Pa HA hydrogel within 2 h. **(D)** Gelation kinetics of the 300-Pa HA hydrogel in 2 h.

A

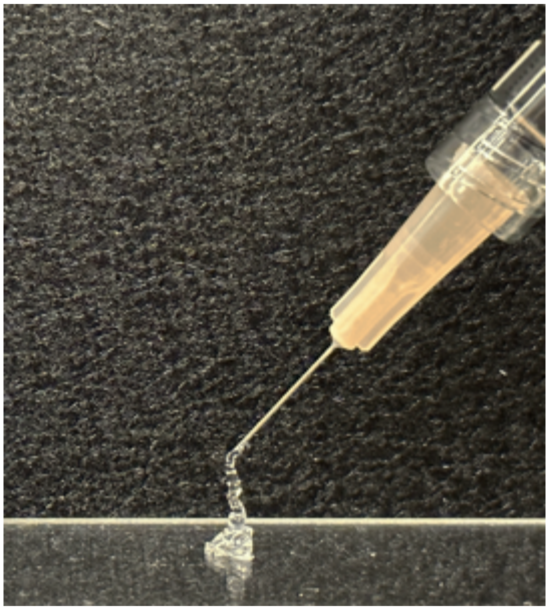

B

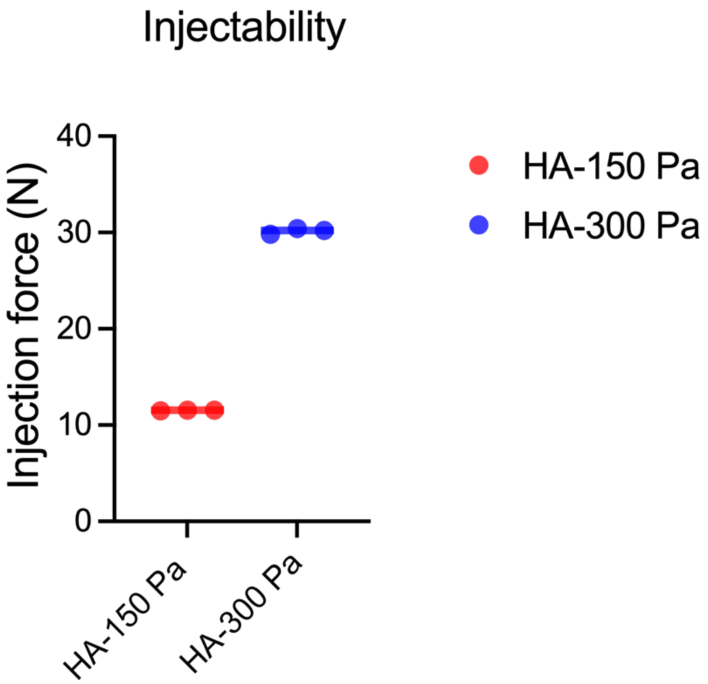

Figure S8. Injection of HA hydrogel
(A) Injection of HA hydrogel through 30-gauge needle. **(B)** Injection forces of the 150-Pa and 300-Pa HA hydrogels. *n* = 3.

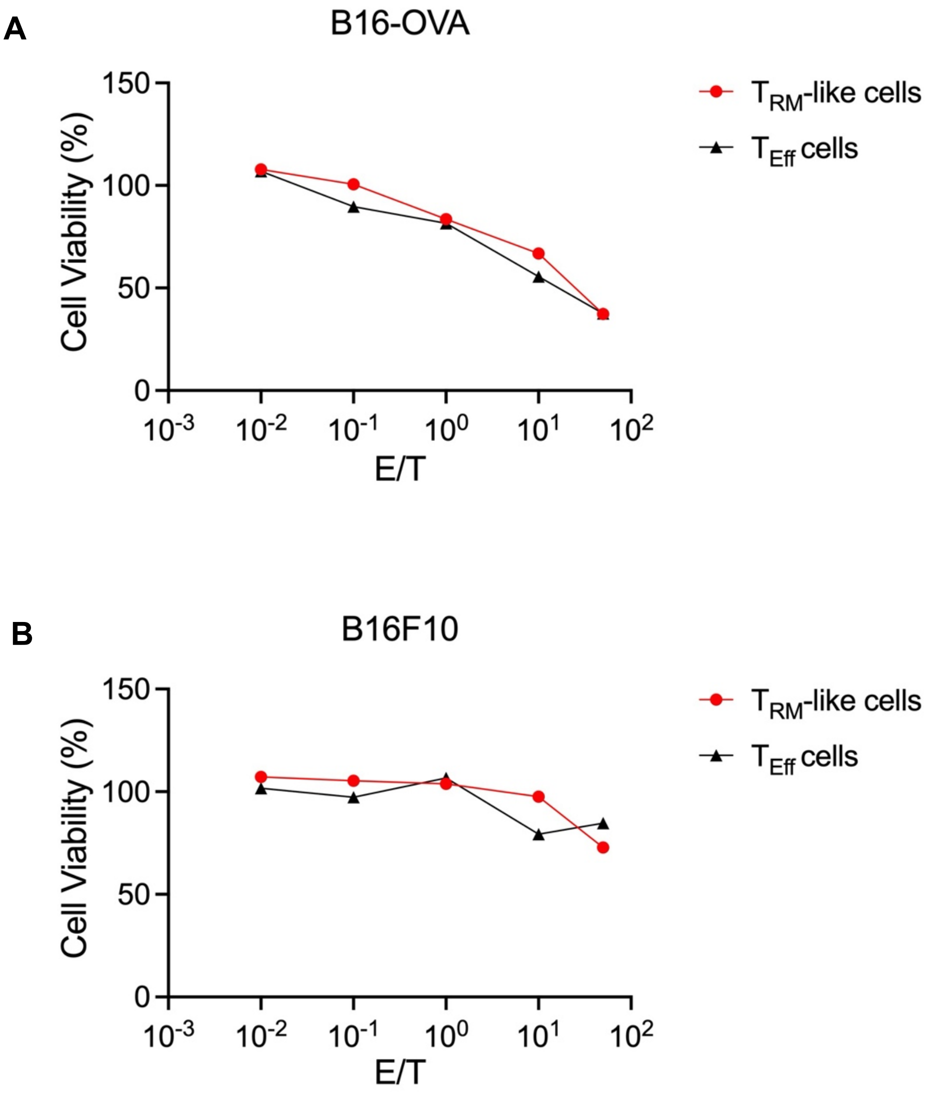

Figure S9. Nano-aAPC stimulated CD8$^+$ T cells induced *in vitro* specific killing.
(A) Specific killing of OT-I effector/effector memory CD8$^+$ T cells or T_{RM}-like CD8$^+$ T cells on B16-OVA tumor cells **(B)** Specific killing of PMEL effector/effector memory CD8$^+$ T cells or T_{RM}-like CD8$^+$ T cells on B16F10 tumor cells. Each bar represents mean ± s.e.m.. n = 3.

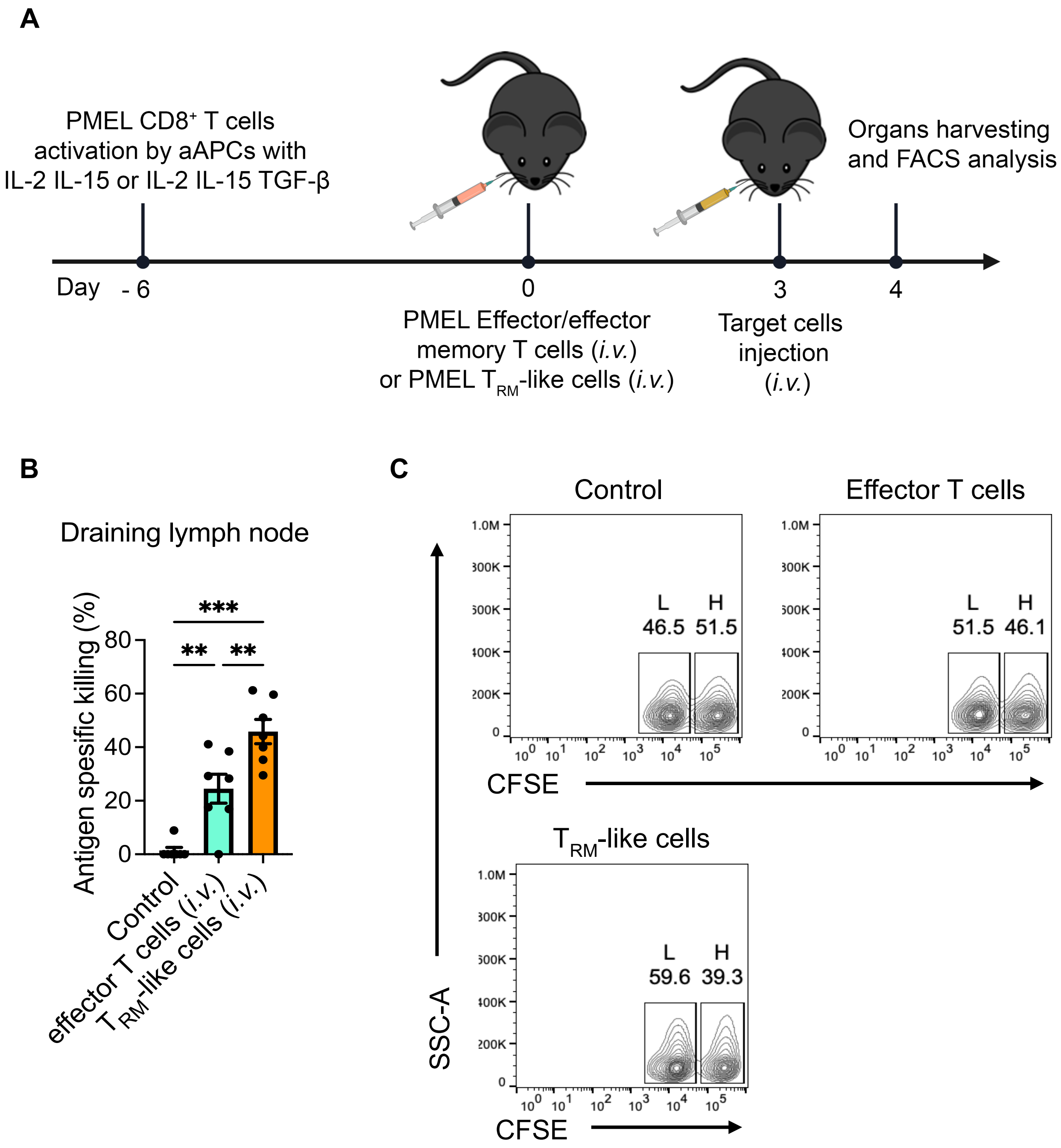

Figure S10. *In vivo* GP100-specific lysis in draining lymph nodes.
(A) Schematic of the experimental setup to access antigen-specific killing of adoptively transferred $CD8^+$ T cells. **(B)** Specific lysis of GP100 pulsed target cells four days after administration with PMEL effector/effector memory $CD8^+$ T cells or PMEL T_{RM}-like $CD8^+$ T cells. Mice were *i.v.* injected with high dose CFSE labeled GP100 peptide-pulsed target cells (CFSE-H) and low dose CFSE labeled control target cells (un-pulsed, CFSE-L) three days post-T cell transfer. Each bar represents mean ± s.e.m.. $n = 7$. One-way ANOVA with Tukey's multiple-comparisons test. $^{**}p < 0.01$, $^{***}p < 0.001$. **(C)** Flow cytometry plots are a representative experiment of 7 replicates.

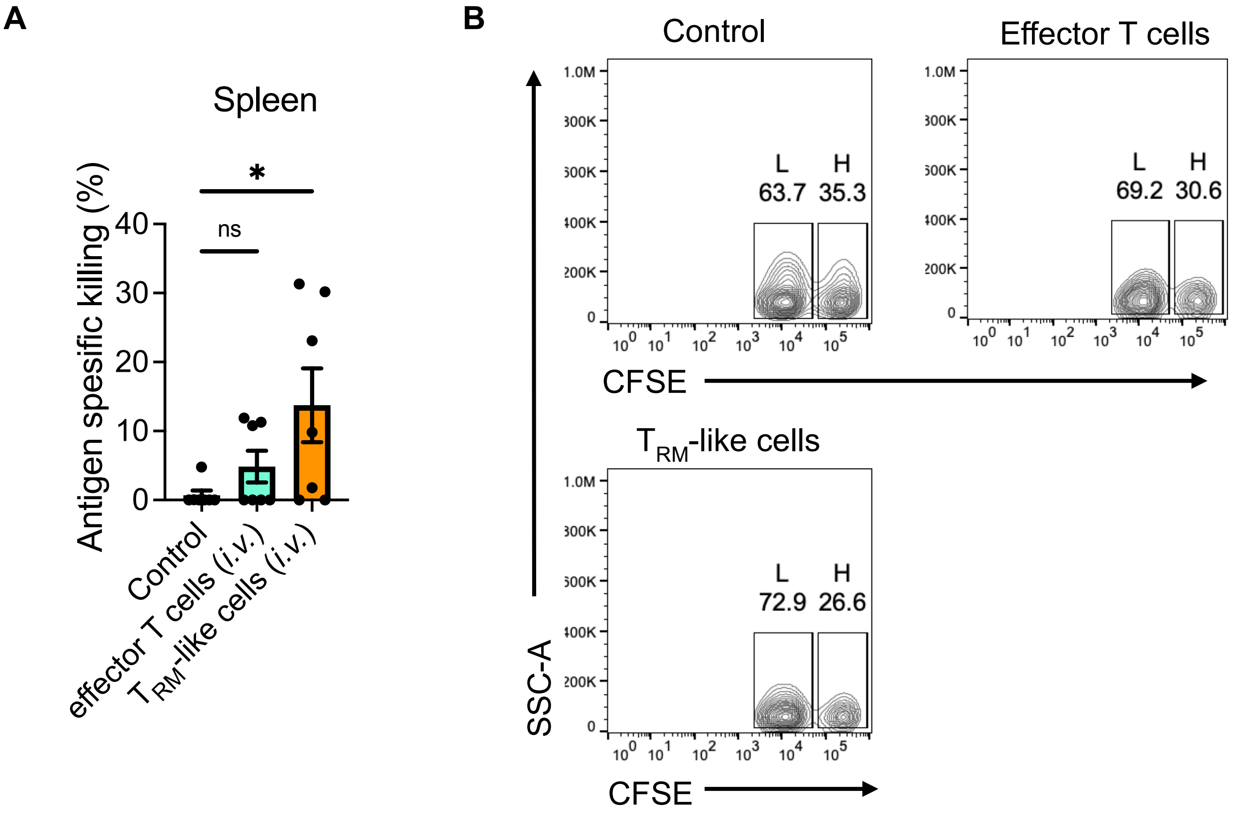

Figure S11. ***In vivo*** **GP100-specific lysis in spleens.**
(A) Specific lysis of GP100 pulsed target cells four days after administration *i.v.* with PMEL effector CD8$^+$ T cells or PMEL T$_{RM}$-like CD8$^+$ T cells. Each bar represents mean ± s.e.m.. $n = 7$. One-way ANOVA with Tukey's multiple-comparisons test. **$p < 0.01$, ***$p < 0.001$. **(B)** Flow cytometry plots are a representative experiment of 7 replicates.

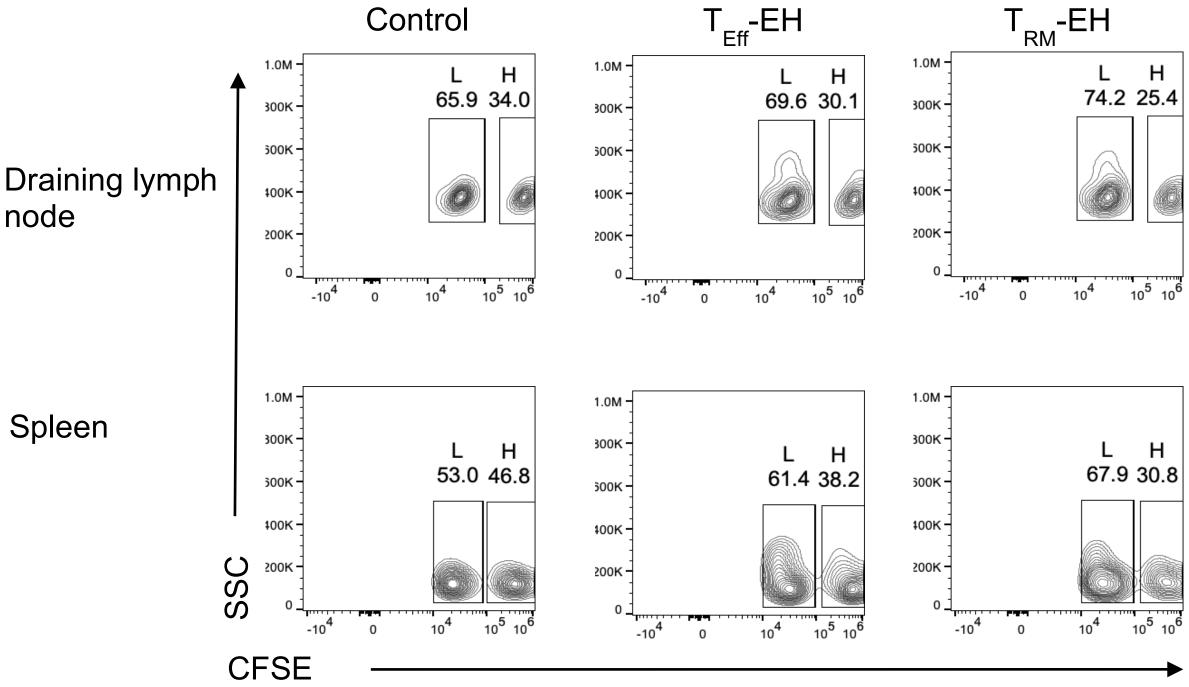

Figure S12. ***In vivo*** **GP100-specific lysis of PMEL T_{Eff}-EH or PMEL T_{RM}-EH in the draining lymph node and spleen.** Flow cytometry plots of *in vivo* GP100-specific lysis in the draining lymph node and spleen for cells collected at 4 days after administration *s.c.* with PMEL T_{Eff}-EH or PMEL T_{RM}-EH.

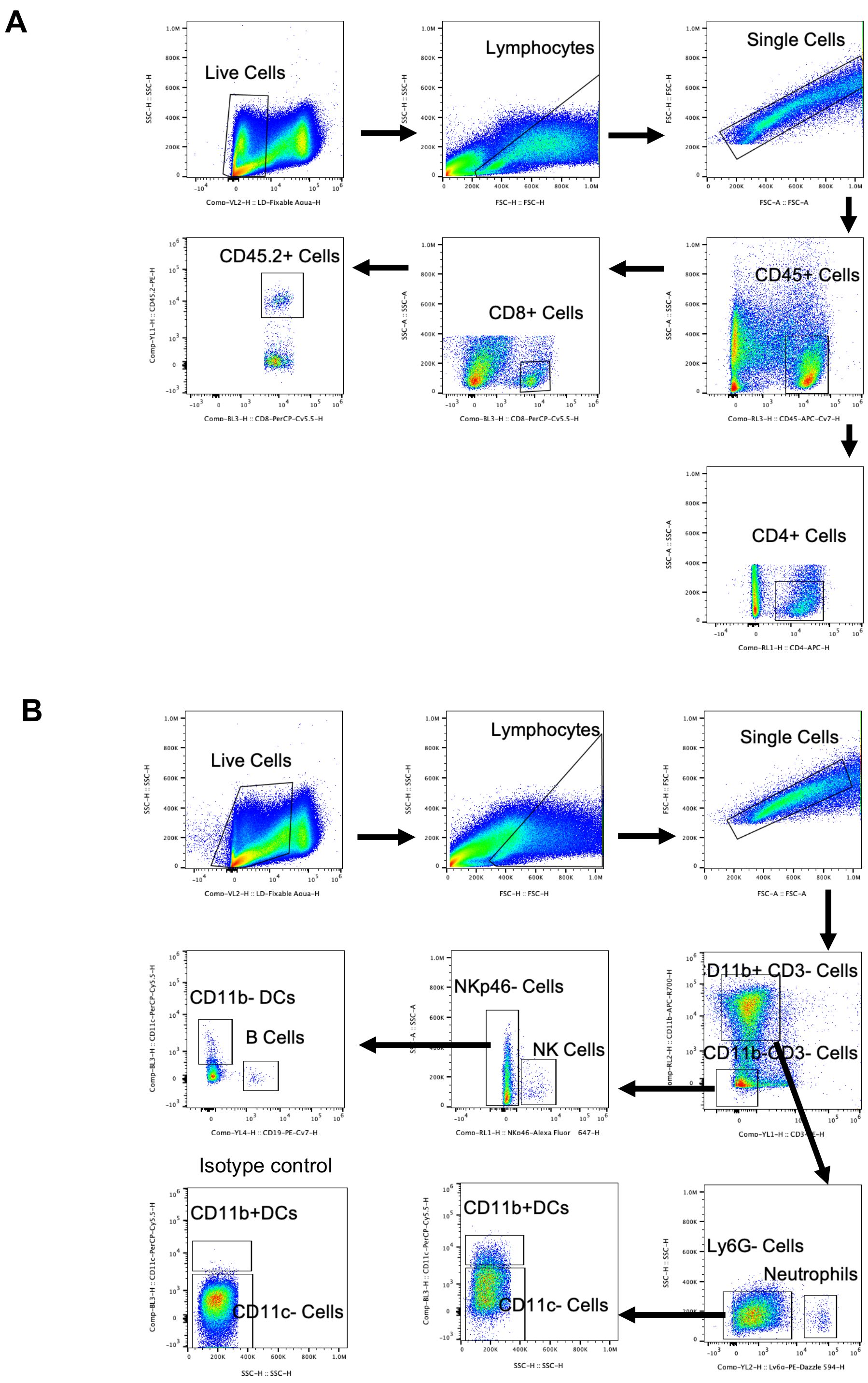

Figure S13. Gating strategy for tumor-infiltrating immune cells analysis.
(A) T cells analysis **(B)** Innate immune cells analysis

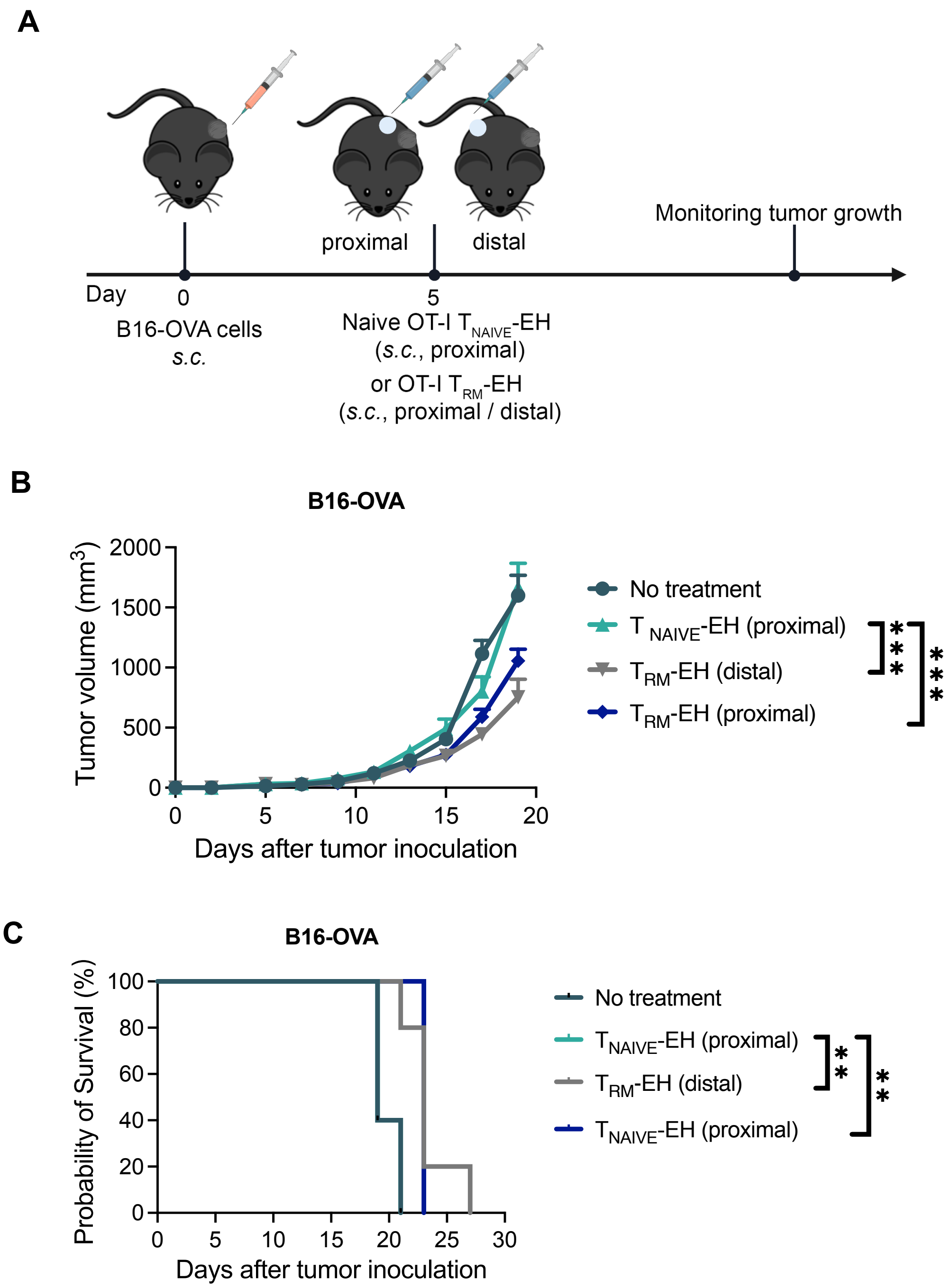

Figure S14. T$_{RM}$-EHs elicited robust local and systemic immune responses in a mouse melanoma model.

(A) Schematic of the experimental design. Mice will be injected *s.c.* with B16-OVA tumor cells. OT-I T$_{RM}$-EHs were *s.c.* injected on the same or opposite tumor flank 5 days post-tumor cell injection. OT-I T$_{NAIVE}$-EHs were injected on the same side of the tumor as a control. **(B)** B16-OVA tumor growth curve with mean ± s.e.m.. $n = 5$. Two-way ANOVA with Tukey's multiple-comparisons test. ***$p < 0.001$. **(C)** Mouse survival curve. $n = 5$. Log-rank test. *$p < 0.05$, **$p < 0.01$.

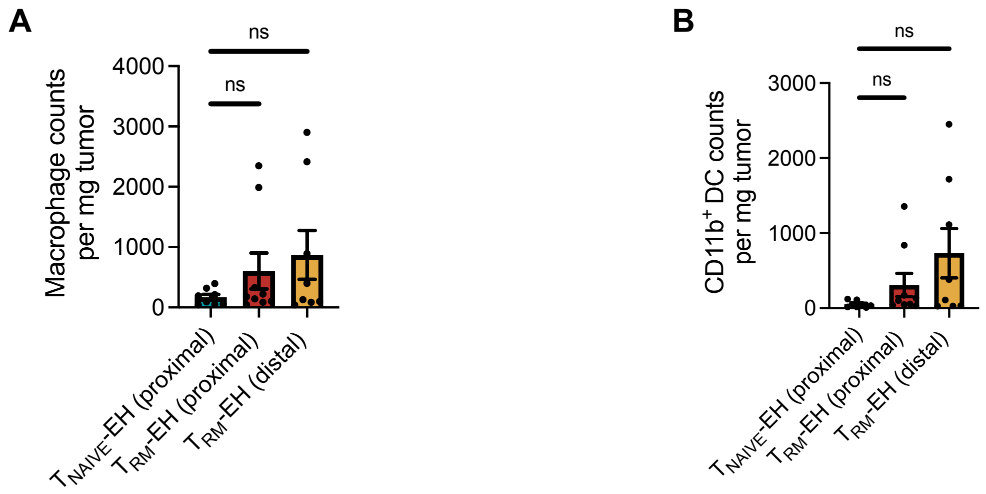

Figure S15. Tumor-infiltrating immune cells analysis in a mouse colon carcinoma model. Quantification of total **(A)** macrophages and **(B)** CD11b^{+} DC cells in the tumor were shown, n = 9, each bar represents mean ± s.e.m.. One-way ANOVA with Tukey's multiple-comparisons test. ns, not significant.

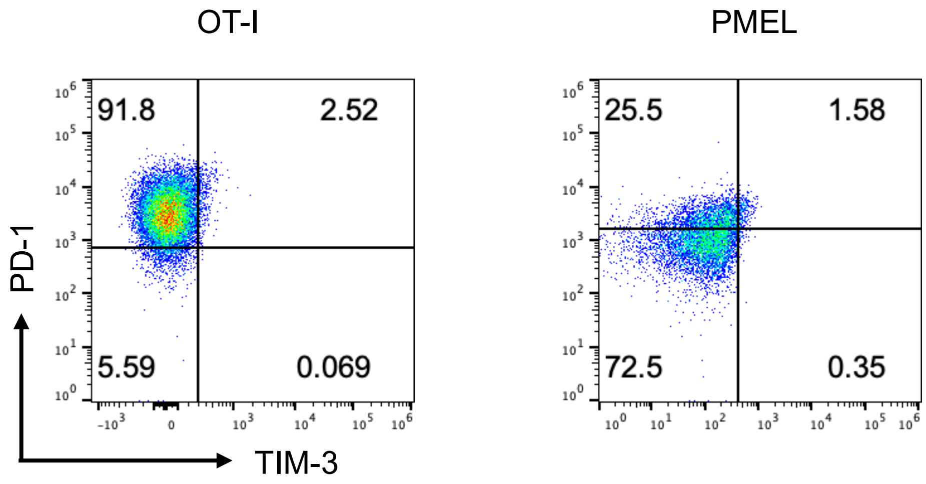

Figure S16. Expression of PD-1 and Tim-3 on OT-I and PMEL T_{RM}-like CD8$^+$ T cells.

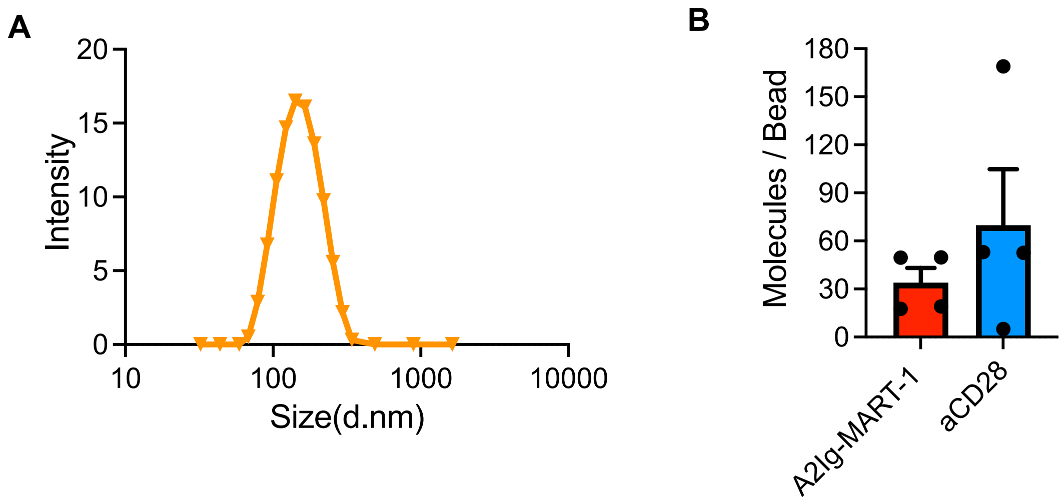

Figure S17. Characterization of A2Ig-MART-1 aAPCs.
(A) Size distribution of A2Ig-MART-1 aAPCs **(B)** Quantification of A2Ig-MART-1 and aCD28 conjugated to aAPCs. Each bar represents mean ± s.e.m.. $n = 4$.

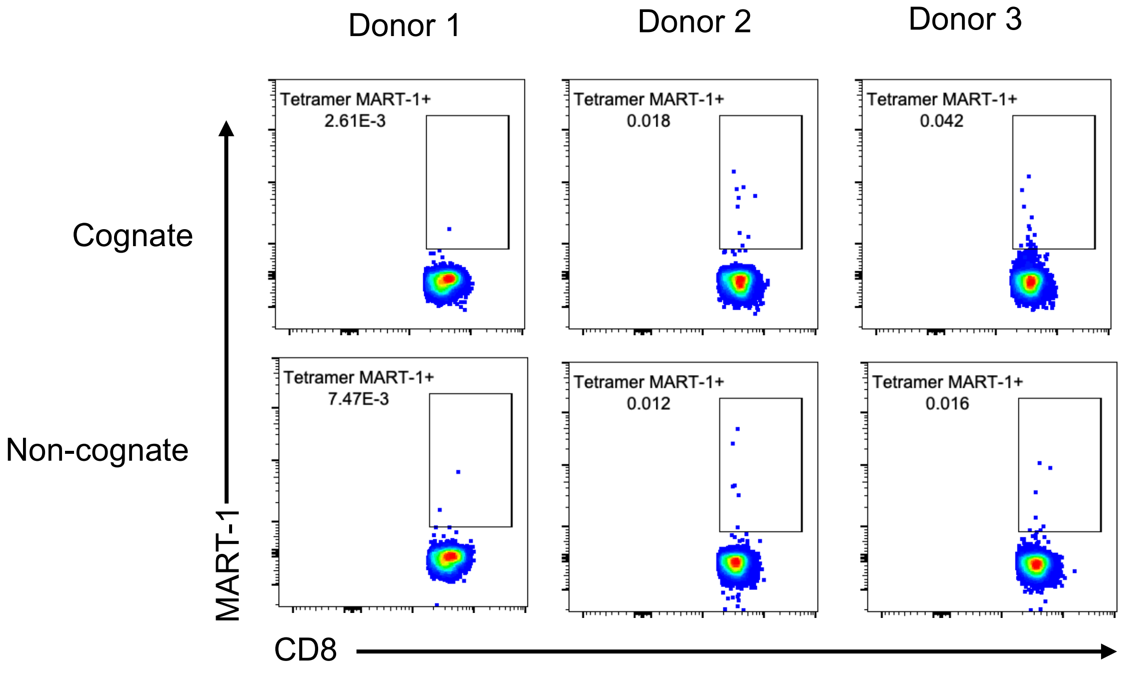

Figure S18. **Tetramer staining of MART-1^{+} CD8^{+} T cells on Day 0 from 3 healthy donors.**

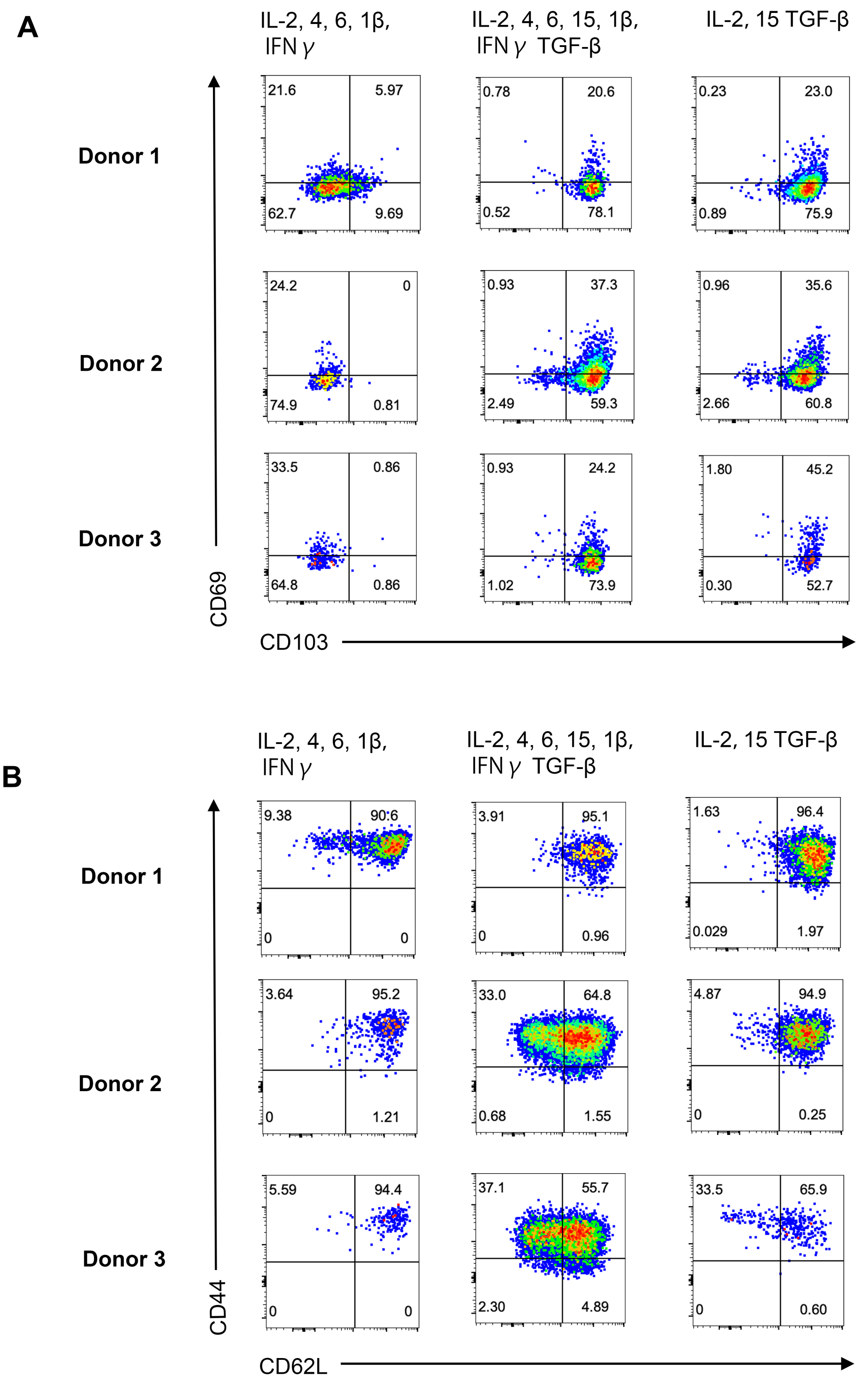

Figure S19. **Expression of T$_{RM}$ marker on MART-1$^+$ CD8$^+$ T cells on Day 14.**
Flow cytometric plots of (A)CD69, CD103 and (B)CD62L expression on MART-1 CD8$^+$ T cells expanded with nano-aAPCs and cultured in different cytokine mixes on Day 14.
